# Cattle on the rocks: Understanding cattle mobility, diet, and seasonality in the Iberian Peninsula. The Middle Neolithic site of Cova de les Pixarelles (Tavertet, Osona)

**DOI:** 10.1371/journal.pone.0317723

**Published:** 2025-01-27

**Authors:** Roger Alcàntara Fors, Richard Madgwick, Laura C. Viñas-Caron, Alexandra J. Nederbragt, Maria Saña Seguí

**Affiliations:** 1 School of History Archaeology and Religion, Cardiff University, Cardiff, United Kingdom; 2 Departament de Prehistòria, Universitat Autònoma de Barcelona, Cerdanyola del Vallès, Spain; 3 University of Copenhagen, Copenhagen, Denmark; 4 School of Earth and Environmental Sciences, Cardiff University, Cardiff, United Kingdom; The University of Winnipeg, CANADA

## Abstract

Reconstructing past herd mobility, reproduction, and diet is crucial for understanding animal management practices among the first sedentary farming communities. It can also shed light on how domestic animals were integrated into the existing exchange networks of goods, products, and raw materials, and how they contributed to broader economic and social changes during the Neolithic. Despite the longstanding importance of cattle (*Bos taurus)* to herders, the role of cattle in the daily, seasonal, and annual cycle of activities of early farming communities remains relatively poorly understood. This study focuses on the Middle Neolithic site of Cova de les Pixarelles (3942–3632 cal. BCE) one of the few sites in the Iberian Peninsula from this period with a substantial collection of faunal remains. The site is particularly notable for its high proportion of cattle remains. Previous research on the cattle bone assemblage from Cova de les Pixarelles has included comprehensive archaeozoological, palaeopathological, and biomechanical analyses—an innovative, integrative approach in Mediterranean archaeology that offers an exceptional level of biographical detail. This study uses bulk bone collagen carbon (δ^13^C_coll_) and nitrogen (δ^15^N) isotopes, and sequential analysis of enamel bioapatite oxygen (δ^18^O) and carbon (δ^13^C_carb_) isotopes to further enhance our understanding of animal management practices during the Middle Neolithic, providing new insights on the diet, mobility and reproduction patterns of cattle. Results indicate that the Neolithic communities that used Cova de les Pixarelles managed these animals to obtain optimal pastures, moving them seasonally from lowland areas to higher mountain plateaus and carefully managing their reproduction cycles. We suggest a nuanced herding approach, combining open-range grazing with an ecological division of herds based on age, sex, and reproductive function, and seasonal vertical mobility, contributing to a complex but efficient herding system during the Middle Neolithic.

## Introduction

Cattle played a crucial role in Neolithic economies. Their versatility, coupled with high meat and dairy productivity, undoubtedly made them a particularly valuable species. Integrating aspects of their feeding, mobility and reproduction presented a significant challenge for early agricultural societies. Recent studies on past cattle herds have started to provide novel insights into different aspects related to their husbandry.

In terms of diet and landscape use, Safoora Kamjan and colleagues [1] showed that at the Neolithic site of Džuljunica (northeastern Bulgaria) (ca. 6200–5500 cal BC), cattle were kept close to the settlement during the summer, in an area with both C3 and C4 plants, while they occasionally fed on forest resources during the winter. In the context of LBK communities (6th millennium BC) in Central Europe, Rosalind Gillis and colleagues [2] documented the provision of seasonal winter fodder, such as leafy hay, and the intensive use of forested areas for grazing. However, some variability can be observed between settlements, with the use of tree fodder at the Bischoffsheim site and the predominance of an herbaceous diet obtained through year-round grazing at Ludwinowo [3]. In the lower Rhine-Meuse Delta during the 4th millennium BCE, cattle herds grazed in open and marshy areas near the settlement, occasionally engaging in winter grazing [4]. In Greece, cattle from Halai and Makriyalos (6th millennium BCE) showed a mixture of C_3_ and C_4_ dietary contributions [5]. During the Late Neolithic, the herds from Makriyalos may have been grazing in coastal marshes located approximately 7 km away from the site. At the Neolithic settlement of Kopydłowo (Polish lowlands), Marciniak and colleagues [6] recorded a shift in the feeding patterns of cattle between the LBK and TRB (Middle Neolithic), with exploitation of increasingly diverse ecological zones and more varied grazing practices. This shift was associated with a transition from collective and communal herd management (LBK) to more individualised and kin-based management (TRB). At the lakeside station of Arbon Bleiche 3, Switzerland (3384±3370 BCE), Gerling and colleagues [7] documented a diversified strategy within the same settlement. Different social units combined local grazing, seasonal movement, and year-round grazing away from the settlement.

As can be seen from these multiple studies, diverse strategies have been documented concerning the provision of food for cattle herds in the past, shaped by factors such as climate, ecology, exploited products, and the type of community organisation. This diversity highlights that animal feeding was a primary concern for early farming communities, as the success of these strategies could determine their subsistence. The varied feeding practices observed in Neolithic cattle herds have, in some instances, been associated with the necessity of extending the annual birthing cycle to ensure a continuous milk supply [2,8,9]. Although the reproductive regimes of *Bos taurus* were also diverse in the Early Neolithic [3,10], in most regions, calving tended to align with seasonal patterns, reflecting climate conditions and forage availability [9].

Based on zooarchaeological and biomolecular studies, the grazing regimes attributed to Neolithic cattle herds encompass a wide spectrum. Strategies implemented range from controlled grazing within the settlement and pasturing in adjacent areas, with or without supplementary feeding [11,12], to forest grazing [13,14], and extensive grazing [15], with or without seasonal movements [16]. In terms of seasonal movements, transhumance and vertical mobility are probably among the most debated and discussed management systems. The concept of vertical mobility is not new, specially within the Mediterranean area [17–21]. However, much focus has been directed to large-scale transhumance movements, usually connected to the intensification of husbandry practices during the Bronze age [18,21–23]. While still within this paradigm, Davidson [24] already pointed out the need to consider the geographical characteristics of the region and the possibilities they offer. Recent analyses [20,25] have focused attention, especially for the Neolithic period, on the concepts of “transterminance” or “valley transhumance” [26], with archaeological evidence supporting the presence of herds and herders in mountainous areas since the Early Neolithic [27–29]. In this context, transterminance is considered to be the seasonal movement of herds from winter pastures in the lower valleys to summer pastures in the higher mountains, always within a radius of 10–25 km [20,25,26,30].

In addition to these strategies, Bogucki’s [31] proposal based on the concept of Open-Range Cattle Grazing offers an alternative point of view. This form of livestock management allows animals to roam freely in search of food rather than being controlled by herders, thus requiring less manual labour than the systems described above. It involves managing herds across a broad landscape instead of confining them to small pastures. Bogucki [31] suggested that this approach might have been used during the Neolithic in the environments where domestic animals were first introduced, implying a form of collective and communal social organisation.

In terms of animal management practices, it is also interesting to consider Tefera’s [32] ecological division of herds, derived from ethnographic studies. According to Tefera, there are two types of herds: “domestic herds” and “camp herds”. The “domestic herd” consists of animals that remain within the settlement, mainly females, that may be bred not only for reproduction but also for milk procurement. The “camp herd” includes surplus animals or those bred for reserve purposes, which are kept in areas with good pasture and water. These herds may move seasonally. The composition of “camp herds” differs from that of “domestic herds”, which may explain the variability in strategies adopted within a single settlement. Factors such as the age and sex of the animals are crucial considerations in characterising the type of grazing practised within a community, along with management, access, and associated use regimes.

It is important to consider that in the Early Neolithic animal husbandry was inherently linked to wider agricultural practices. For example, the production of fodder or supplementary feed for cattle, the rotation and nutritional diversity of pastures, and the potential for overexploitation of land and pasture, are important aspects influencing herd health. These factors depend on the complementarity of agricultural and livestock cycles and their adaptability to local climatic and ecological conditions. The choice of cattle grazing models is contingent on a variety of factors, including climate, land and resource availability, herd composition, and production goals, thereby conditioning Neolithic animal husbandry practices. Proper management of animal nutrition was critical for maintaining herd health and ensuring the sustainability of pastures and croplands.

Furthermore, strontium isotope analyses have revealed mobility patterns that could be related to the circulation or exchange of cattle during the Neolithic, as demonstrated in the study by Sjögren and Price [33] at the Falbygden site, in Sweden. This study suggests widespread domestic animal circulation in the western area of Sweden, although long-distance movements were not documented. This shows that not all movements are necessarily directly related to grazing activities [34,35].

In the Iberian Peninsula, cattle husbandry is documented from the earliest moments of the Neolithic (5600–5000 cal. BCE). The relative importance of cattle increases steadily throughout the Neolithic, although it does not completely overshadow the importance of sheep [36]. In some Early Neolithic sites, their economic weight is remarkable, exceeding 30% [37]. Cattle herds appear to be of greater relative importance in lowland open-air sites than in cave and highland sites [20]. While meat exploitation seems to have been the primary objective of cattle management, there is also evidence for milk production [38] and use of their labour force [39,40]. From the Middle Neolithic onwards, their exploitation becomes even more extensive, exceeding 80% in some faunal assemblages.

One of the most important sites is Cova de les Pixarelles (3942–3632 cal BCE), which is the main focus of this work. The Middle Neolithic layer of Cova de les Pixarelles provides a unique opportunity to explore issues related to the adoption of cattle as domestic animals and their economic importance for Neolithic farming communities. The development of specific management strategies and the exploitation of new ecological niches are key issues to address.

The main objective of this study is to comprehensively characterise the management of one of the earliest Neolithic cattle herds inhabiting ecologically defined areas characterised by a rugged topography. This herd was exploited in a specialised way, and its composition is well-documented. The aim is to achieve a high level of explanatory resolution regarding the initial livestock management of this species and its ecological adaptation.

Thus, this paper investigates past management strategies, including diet, environment, seasonal mobility, and birth season, of the cattle herd population from the Middle Neolithic Cova de les Pixarelles (3942–3632 cal BCE) using a combination of bulk bone collagen carbon (δ^13^C_coll_) and nitrogen (δ^15^N) isotopes and sequential analysis of enamel bioapatite oxygen (δ^18^O) and carbon (δ^13^C_carb_) isotopes. To complement the interpretation of the obtained results, cattle bone remains from the northeastern Iberian Peninsula archaeological sites of La Draga (Banyoles, 5324–4980 cal BCE) [41] and Reina Amàlia (Barcelona, 4670–4463 cal BCE) [42] were analysed as a reference. Both sites, with a significant presence of bovines in their faunal assemblages, belong to the Early Neolithic. The combined results will provide new insights into the diversity of management strategies developed for one of the earliest Neolithic cattle herds in the Iberian Peninsula and its adaptation to diverse ecological areas.

## Materials and methods

### Cova de les Pixarelles: The site and the zooarchaeological assemblage

**Fig 1:**
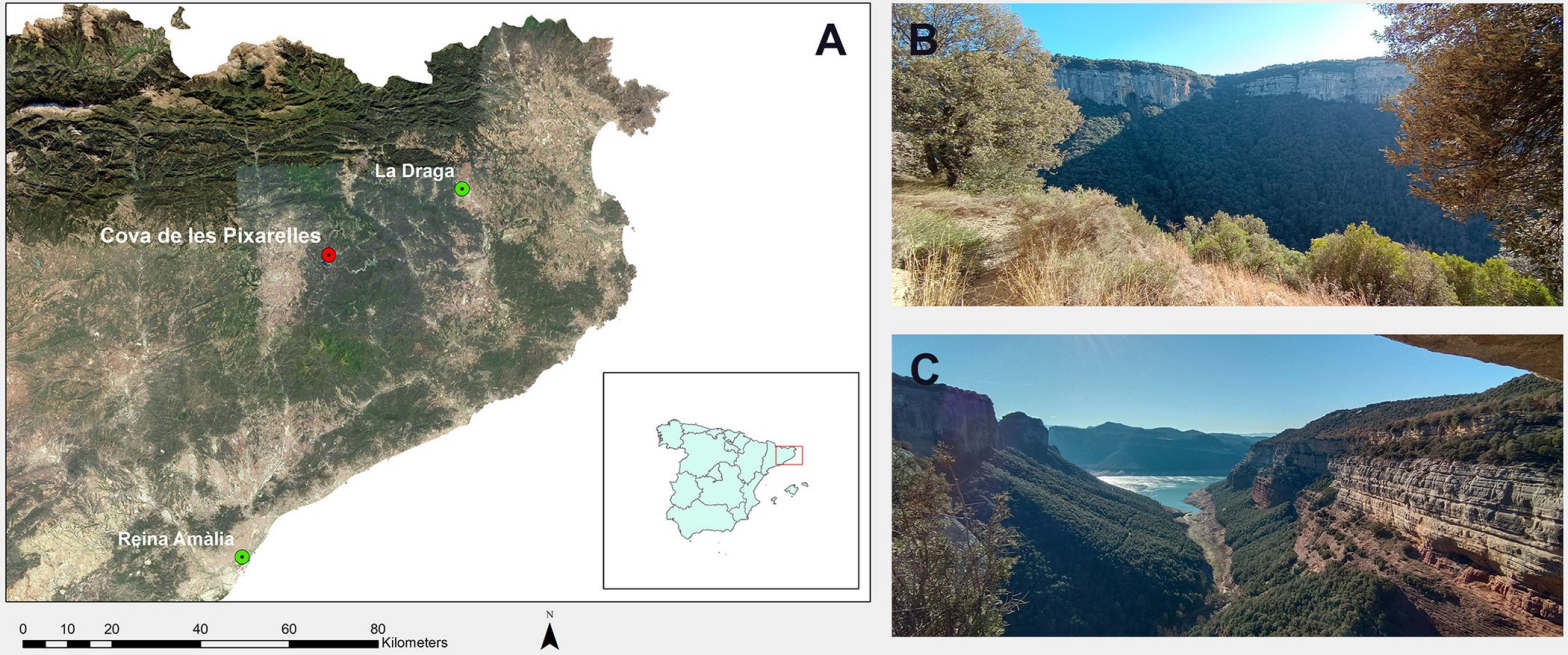
Location and landscape of Cova de les Pixarelles. (A) Background map of Catalunya derived from the WMS Territorial Ortophoto of the Institut Cartogràfic i Geològic de Catalunya (ICGC), used under a CC BY 4.0 license, accompanied by the Municipal, provincial and autonomous boundaries of Spain (CC-BY 4.0 ign.es) (B) View from the base of Cova de les Pixarelles facing south towards the Balà Stream and Cingles de Balà (Balà cliffs). Credits of the photo to R. Alcàntara.(C) On the way to Cova de les Pixarelles, view of the Balà Stream from Morro de l’Abella at its confluence with the Sau Reservoir. Credits of the photo to R. Alcàntara.

Cova de les Pixarelles is a cave site located in the natural region of Collsacabra (Fig 1). This mountainous region is characterised by high plateaus, steep cornices and abrupt slopes, mainly due to the erosion of water courses such as the Ter River and its tributary the Balà stream. The cave is located at an altitude of 670 m asl, in the middle of the vertical wall of the plateau facing the Balà stream. The site contains multiple occupation levels, with the most significant layers corresponding to the Chalcolithic, Late Neolithic, and Middle Neolithic periods. These layers are sealed by calcareous deposits, which contributed to the site’s good preservation [43–45]. The Middle Neolithic layer (3942–3655 cal BCE and 3763–3632 cal. BCE, 95.4%) (Re-calibrated from Alcàntara Fors [40] using OxCal v4.4.4 [46,47]; Atmospheric data from Reimer and colleagues [48], IntCal 20 calibration curve) was documented in a stratigraphic survey of 6 m^2^ [49]. This layer is characterised by a dark-grey sediment rich in ashes and charcoal, abundant faunal remains, and fewer ceramic fragments and lithic tools [49]. Soil micromorphology studies identified a more complex micro-stratigraphy, identifying three major microfacies. These microfacies show a sequence of fire events: initially, interrupted fires used for cooking, followed by sustained fires used for lighting, cooking, hygiene, or meat smoking, with evidence of high-intensity combustion [49]. This evidence, together with the lack of plant phytoliths and spherulites and the absence of coprolites, argue against the use of the cave for stabling or penning, as has been documented in other Neolithic sites around the Iberian Peninsula [50–55]. The archaeological elements recovered, as well as the sedimentation dynamics of the Middle Neolithic layer, are evidence of its rapid formation [49]. Based on the context and the nature of the archaeological remains, it is reasonable to conclude that the multiple events that contributed to the formation of the layer occurred within a relatively short time [56]. The presence of well-preserved organic remains, such as bones, ashes and charcoal, suggests that the layer was sealed relatively quickly after deposition, preventing significant post-depositional disturbance [56]. The fact that the occupations appear sealed by calcareous concretions, which are indicative of the cave’s natural stratification process, suggests that the periods of occupation and abandonment were cyclical and potentially related to the seasonal use of the cave. This could imply that the depositional events were clustered in time, perhaps corresponding to specific seasons or activities that brought people to the cave regularly [49].

In terms of animal resources management, Cova de les Pixarelles represents an unusual, almost unique site for the entire Neolithic of the Iberian Peninsula. All bone remains were recovered, precisely recorded with coordinates, and photographed during excavation. The recovery of faunal remains was exhaustive, combining manual collection with flotation and sediment sieving to minimize bias. All the faunal remains recovered have been analysed. The Middle Neolithic faunal assemblage comprises 456 specimens (NISP = 366), of which 292 have been identified as *Bos taurus* [40].

All remains are from domestic animals. Based on the NISP, 80% correspond to cattle, 17% to sheep/goat and 3% to pig [40]. Cattle also dominate the assemblage when considering the minimum number of elements (MNE) and minimum number of individuals (MNI) [40]. Based on bone fusion and teeth wear stages, these remains represent a minimum number of individuals (MNI) of seven cattle [40]. Archaeozoological analysis evidenced a prevalence of meaty parts of the fore and hindlimbs, though all skeletal parts are represented (S1 Table). The age profile demonstrated an abundance of adult and subadult specimens [40] (S1 and S2 Figs). These characteristics, common to all species represented, suggest that these animals were exploited for their meat. Size-wise, the cattle from Cova de les Pixarelles clearly fall in the range of domestic cattle, rather than aurochs [40]. The assemblage appears to be comprised of males and females, but sexing evidence is mostly limited to linear measurements and a clear separation of the specimens could not be achieved due to the large size overlap (S3 Fig). Given the composition of the assemblage and the characteristics of other categories of archaeological remains recovered in this level, it has been suggested that Cova de les Pixarelles is a place used for specific activities and not as a permanent residence [40,56]. The pottery assemblage consists of fragments of small and medium-sized vessels (open bowls and plates, and ovoid and cylindrical vessels with rounded bottom), with a homogeneous, low temperature firing and a general lack of decorative elements. These vessels were used as kitchenware, probably for the preparation and transformation of food, to contain small quantities of liquids and other foodstuffs. The lithic assemblage can be related to similar functions. The most outstanding elements are a cornubianite axe and a series of macrolithic hand tools (7–10 cm) made from local raw materials [56,57]. Finally, the significant presence of high-meat-content skeletal parts and diaphysis with fractures made when the bones were still fresh suggests that the cave was probably used as a place to obtain and prepare food [57].

With this in mind and aiming to assess whether the herd was stabled at the site or in its immediate vicinity, the cattle remains from Pixarelles have been examined from a number of perspectives. Recent biomechanical analyses of the first phalanges revealed a relatively high level of activity of the animals with load-bearing forces generally more intense on the hind limb, suggesting mobility on a consistently uneven terrain [40]. Palaeopathological examination of the bones revealed healing rib fractures and tooth loss with resorbed alveoli that suggesting a relatively traumatic lifestyle [58,59]. Both studies provide complementary evidence that cattle from Cova de les Pixarelles probably moved in and around the rocky region of Tavertet, where the cave is located.

### Materials

Sample selection was based on the total number of *Bos taurus* bones recovered. Considering the composition and condition of the assemblage, and taking into account the established MNI, five cattle specimens from the Middle Neolithic layers of Cova de les Pixarelles were selected for sequential enamel oxygen (δ^18^O) and carbon (δ^13^C_carb_) isotopic analysis. The sample consists of 5 mandibles from which the second and third molars were selected. In four cases the teeth come from complete mandibles (PIX1, PIX2, PIX4 and PIX5) and in one case the teeth are isolated and correspond to the same mandible (PIX3). Additional samples were taken from the mandibles for carbon (δ^13^C_coll_) and nitrogen (δ15N) isotopic analysis of bone collagen to provide complementary dietary information. Root dentine collagen from M2 was used for the isolated teeth.

To better contextualize the δ13C and δ15N results from the mandibular samples, a total of twenty additional cattle bone samples were collected from the same site, along with samples from two other Early Neolithic sites in the northeastern peninsula—La Draga (5324–4980 cal BC) [41] and Reina Amalia (4670–4463 cal BC) [42]—which have good representation of this species. Twenty-five samples were taken from La Draga and six from Reina Amalia (S3 Table). Previously published data from these sites [60] have also been included in the plots and tables corresponding to the comparative analyses. The choice of these sites was also motivated by the fact that they are located in environments with different characteristics. In the case of Draga, it is a lacustrine environment (Banyoles lake), while Reina Amalia is located on the coastal plain (Barcelona).

### Methods

The method implemented combines archaeozoological analysis, sequential tooth sampling and bone collagen sampling. The faunal samples analysed in this study were recovered during archaeological excavations at the sites of Cova de les Pixarelles, La Draga, and Reina Amàlia authorised by the competent agency of the Generalitat de Catalunya (Servei d’Arqueologia i Paleontologia de la Generalitat de Catalunya) and were analysed in the framework of ongoing research projects (Deparatment of Prehistory, UAB), with the consent of the directors of the archaeological excavation projects.

### Carbon (δ^13^C_coll_) and Nitrogen (δ^15^N)

For δ^13^C and δ^15^N isotope analysis of the Pixarelles mandibles, the collagen extraction protocol followed a modified Longin method [61]. A fragment of 0.5 to 1 g of bone was sampled from each specimen using a Dremel with diamond wheel attachment and abraded using a diamond burr to remove any superficial contaminants and the outer c. 20 μm of bone. Fragments were then demineralised in 0.5M hydrochloric acid (HCl) at 5°C, with acid being changed every three days until demineralisation was complete (typically 7 days). Demineralised samples were rinsed three times with deionised water and gelatinised in the heating block at 75°C for 48 hours in pH3 H_2_O, acidified using HCl. The gelatinised collagen was separated from any residual fraction using Ezee filters and transferred to polypropylene tubes for freezing. The samples were then freeze-dried. Collagen (0.9±0.2 mg) was weighed in 6x4 tin capsules and analysed in duplicate using a Thermo Delta V Advantage IRMS coupled with a Flash EA CN analyser at the Stable Isotope Facility at Cardiff University The baseline bone remains from Cova de les Pixarelles, La Draga and Reina Amàlia was prepared at the Autonomous University of Barcelona and analysed at ICTA-UAB. Collagen extraction followed the same protocol, but gelatinised collagen was ultrafiltered before frozen and freeze-dried.Isotope ratios are reported as δ^13^C and δ^15^N, relative to internationally defined scales, Vienna Pee Dee Belemnite (VPDB) for carbon isotopes, and atmospheric air (AIR) for nitrogen isotopes, expressed per mil (‰). Atomic C:N ratios complied with collagen quality control criteria for nitrogen and carbon [62]. Carbon and nitrogen isotope ratios were calibrated against in-house caffeine (laboratory grade, 98.5%, Acros Organics) and a marine collagen standard, as well as international standards IAEA-600 (δ^13^C and δ^15^N), IAEACH-6 (δ^13^C) and IAEA-N2 (δ^15^N). The 1σ (n = 55) standard reproducibility was ±0.06 for δ^13^C and ±0.07 for δ^15^N.

All five mandible samples from Cova de les Pixarelles produced collagen and complied with quality control standards for atomic C:N ratios in δ15N and δ13C [62]. Regarding the baseline bone samples from the same site, four did not yield collagen, and five exceeded the acceptable C:N ratio, excluding them from analysis. At Reina Amàlia, one sample failed extraction, and one exceeded the C:N ratio limit. At La Draga, collagen was absent in eight samples, while the remaining samples met quality control standards.

### Oxygen (δ18O) and carbon (δ13C_carb_)

To evaluate the periodic variation of δ^18^O and δ^13^C, tooth enamel was sequentially sampled following established protocols [63]. A total of 154 samples were extracted from the buccal side of the hypoconid lobe of M2 and M3 after gently abrading the surface with a tungsten drill to access the enamel and remove potential contaminants. Horizontal, 1 mm sample bands were extracted at intervals of 1 to 1.5 mm from the apex to the enamel-root junction (ERJ). Sample position was measured from the ERJ.

Samples were then acidified for 5 minutes with >100% ortho-phosphoric acid at 70°C. The δ^18^O and δ^13^C composition of the sample powders was measured on a Thermo Mat 253 dual inlet mass spectrometer coupled to a Kiel IV carbonate preparation device at the Stable Isotope Facility at Cardiff University. Results are reported per mil (‰) relative to the Vienna Pee Dee Belemnite (VPDB) scale. In-house standards are calibrated against NBS 19. Using NBS19 as a single anchor point, the measured δ^18^O values for NBS18 are within one standard deviation of the accepted values. The long-term precision of an in-house Carrara marble standard is ≤0.04‰ in δ^18^O and ≤0.03‰ in δ^13^C.

The δ^18^O sequences were modelled to standardise and minimise inter-individual variability using an equation based on a cosine function following Balasse and colleagues [64]. Mean of the Squared Error (<0.03) and Pearson´s correlation values (>0.9) are provided as measures of the fitness of the model. To this end the equation uses the amplitude of the curve (A, in ‰), the distance from the enamel-root junction of the maximum value of δ^18^O (x_0_, in mm), the value for the annual growth of the tooth (X, in mm) and the mean of the maximum and minimum value (M, in ‰). The x_0_/X ratios obtained vary with the season of birth from 0 to 1 and can be constrained within an annual cycle, providing a measure of the length of the period of births and timing. Consequently, the values are represented in a circular graph divided into 12 parts, as a virtual representation of the months of the year and seasonal events. Contrary to sheep, reference x_0_/X ratios of controlled modern cattle are not available, and thus interpretation is based upon the available data regarding cattle and auroch birth seasonality in natural and anthropogenic contexts (*e*.*g*. [1,8,9,65–69]). The One-way ANOVA test has been used to evaluate significant differences between mean values of δ^18^O and δ^13^C sequences.

## Results

### Carbon (δ^13^C_coll_) and nitrogen (δ^15^N)

The δ^13^C and δ^15^N values for Cova de les Pixarelles’ mandibles are presented in Table 1. All five samples provided good collagen yields, with *δ*^13^C and *δ*^15^N percentages and atomic C:N ratios located within the accepted ranges [70–72]. The C:N ratios for PIX3 fall very narrowly outside (0.04‰) of the more stringent ranges put forward by Guiry and Szpak [73] and this may have a negligible effect on the data.

**Table 1:** δ15N and δ13C results for the mandibles from Cova de les Pixarelles. All values represent the mean of the duplicates.

ID	Element	δ^15^N (‰ AIR)	σ	δ^13^C (‰ VPDB)	σ	%N	%C	C:N	Yield (%)
PIX1	Mandible	4.7	0.01	-19.9	0.03	9.9	27.6	3.26	25.9
PIX2	Mandible	4.1	0.01	-20.1	0.02	14.0	39.1	3.27	1.9
PIX3	M2 root	4.4	0.07	-20.3	0.01	15.4	43.9	3.33	1.4
PIX4	Mandible	3.9	0.04	-20.6	0.20	11.7	33.1	3.24	9.7
PIX5	Mandible	5.1	0.08	-19.4	0.07	13.1	36.9	3.27	1.6

Both δ^13^C and δ^15^N values exhibit low variability. δ^13^C values range from -20.6‰ to -19.4‰ (M = -20.1‰, σ = 0.5) and indicate a clear dietary reliance on C_3_ terrestrial resources. δ^15^N values range from 3.9‰ to 5.1‰ (M = 4.5‰, σ = 0.5). There are no marked outliers and both δ^13^C and δ^15^N values are within a total range of 1.2‰.

**Fig 2:**
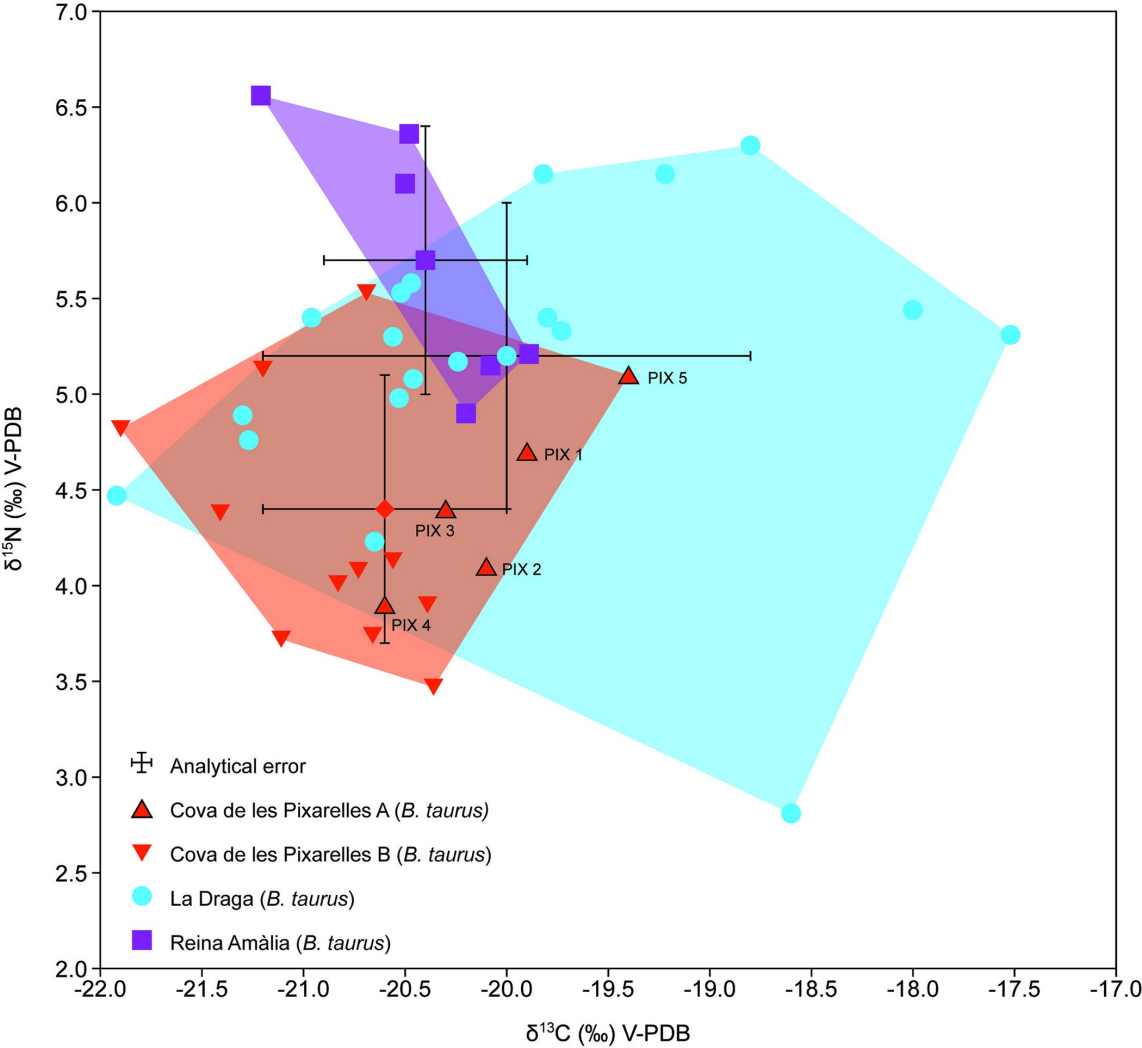
Scatter plot with the δ^13^C and δ^15^N values of *Bos taurus* from Cova de les Pixarelles, La Draga and Reina Amàlia. The dataset includes the results of *Bos taurus* mandible samples from Cova de les Pixarelles (n = 5) and *Bos taurus* baseline values from the sites of Cova de les Pixarelles (n = 11), La Draga (n = 19) and Reina Amàlia (n = 4) prepared for this study and previously published values [99] from La Draga (n = 2) and Reina Amàlia (n = 2). The polygons represent the bivariate range for each dataset.

The detailed results obtained for the baseline can be consulted in S3 Table. Samples from Cova de les Pixarelles present similar values to the mandibles, ranging from -21.9‰ to -20.4‰ (M = 20.9‰, σ = 0.5) for δ^13^C values, and from 3.5‰ to 5.5‰ (M = 4.3‰, σ = 0.6) for δ^15^N values. In contrast, La Draga presents broad variability in both δ^13^C and δ^15^N. δ^13^C values range from -21.9‰ to -17.5‰ (M = -20.0‰, σ = 1.2) and δ^15^N range from 2.8‰ to 6.3‰ (M = 5.2‰, σ = 0.8). Reina Amàlia presents more uniform δ^13^C values, which range from -21.2‰ to -19.9‰ (M = -20.4‰, σ = 0.5), and nitrogen values (Fig 2, Tables 2 and 3).

**Table 2:** Summary statistics of δ^13^C for the specimens analysed in this work and the baseline values.

		C. de les Pixarelles A	C. de les Pixarelles B	C. de les Pixarelles AB	Reina Amàlia	La Draga
**δ** ^**13**^ **C**	N	5	11	16	6	19
	Min	-20.6	-21.9	-21.9	-21.2	-21.9
	Max	-19.4	-20.4	-19.4	-19.9	-17.5
	Mean	-20.1	-20.9	-20.6	-20.4	-20.0
	Std. error	0.2	0.1	0.2	0.2	0.3
	Variance	0.2	0.2	0.4	0.2	1.3
	Stand. dev	0.5	0.5	0.6	0.5	1.2

C. de les Pixarelles A refers to the samples taken from Cova de les Pixarelles’ mandibles and teeth and analysed at Cardiff University. C. de les Pixarelles B are the samples from Cova de les Pixarelles measured as a baseline at the Autonomous University of Barcelona. C. de les Pixarelles AB reflects the joint variability of these two groups of samples. Reina Amàlia and La Draga include the baseline samples prepared for this study and two each from published sources [74].

**Table 3:** Summary statistics of δ^15^N values for the specimens analysed in this work and baseline values.

		C. de les Pixarelles A	C. de les Pixarelles B	C. de les Pixarelles AB	Reina Amàlia	La Draga
**δ** ^**15**^ **N**	N	5	11	16	6	19
	Min	3.9	3.5	3.5	4.9	2.8
	Max	5.1	5.5	5.5	6.6	6.3
	Mean	4.4	4.3	4.3	5.7	5.2
	Std. error	0.2	0.2	0.1	0.3	0.2
	Variance	0.2	0.4	0.3	0.5	0.6
	Stand. dev	0.5	0.6	0.6	0.7	0.8

C. de les Pixarelles A refers to the samples taken from Cova de les Pixarelles mandibles and teeth for this study and analysed at Cardiff University. C. de les Pixarelles B are the samples from Cova de les Pixarelles measured as a baseline at the Autonomous University of Barcelona. C. de les Pixarelles AB reflects the joint variability of these two groups of samples. Reina Amàlia and La Draga include the baseline samples prepared for this study and two each from published sources [74].

In the case of the δ^13^C values, it is interesting to highlight the overlapping ranges of the three sites. When comparing Cova de les Pixarelles, Reina Amàlia and La Draga using a One-Way ANOVA, no significant differences were observed in δ^13^C values (F value = 2.072; p = 0.14). However, four of La Draga samples stand out for their higher δ^13^C values, with values enriched between 0.5‰ and 2.2‰ compared to the next highest value (-19.7‰). Differences in δ^15^N values were statistically significant (F value = 10.97; p = 0.000173). In particular, Tukey pairwise-comparisons indicated no differences between Reina Amàlia and La Draga (p = 0.2379949) but showed that Cova de les Pixarelles had significant lower δ^15^N values compared to La Draga (p = 0.0026154) and Reina Amàlia (p = 0.0005056). Comparing the means with Cova de les Pixarelles, La Draga values are enriched by 0.9‰, and Reina Amàlia´s by 1.4‰.

### Oxygen (δ^18^O) and Carbon (δ^13^C_carb_)

The δ^18^O and δ^13^C results for the five mandibles of Cova de les Pixarelles are summarised in Tables 4 and 5 and can be consulted in detail in S4 Table.

**Table 4:** Descriptive statistical values of δ^18^O sequences of M2 and M3 for all specimens (PIX1–5).

		PIX 1		PIX 2		PIX 3		PIX 4		PIX 5	
		M2	M3	M2	M3	M2	M3	M2	M3	M2	M3
**δ** ^**18**^ **O**	N	15	18	13	19	19	17	13	17	8	15
	Min	-6.4	-7.2	-6.4	-6.9	-7.6	-7.9	-6.3	-6.2	-5.9	-5.6
	Max	-5.5	-5.5	-5.2	-4.7	-4.7	-5.5	-5.2	-4.8	-4.0	-4.4
	Mean	-6.0	-6.5	-5.7	-5.9	-6.4	-6.4	-5.9	-5.5	-5.2	-4.8
	Std. error	0.07	0.14	0.09	0.14	0.21	0.18	0.09	0.10	0.22	0.08
	Variance	0.07	0.33	0.11	0.37	0.82	0.52	0.10	0.18	0.40	0.10
	Std. dev	0.26	0.57	0.33	0.61	0.91	0.72	0.32	0.43	0.63	0.32

**Table 5:** Descriptive statistical values of δ^13^C sequences of M2 and M3 for all specimens (PIX1–5).

		PIX 1		PIX 2		PIX 3		PIX 4		PIX 5	
		M2	M3	M2	M3	M2	M3	M2	M3	M2	M3
**δ** ^**13**^ **C**	N	15	18	13	19	19	17	13	17	8	15
	Min	-11.2	-11.2	-11.1	-10.9	-12.3	-11.1	-11.6	-11.3	-11.4	-11.7
	Max	-9.9	-10.0	-10.5	-10.2	-10.7	-10.0	-11.0	-9.7	-9.9	-9.9
	Mean	-10.8	-10.7	-10.8	-10.6	-11.4	-10.6	-11.4	-11.0	-10.7	-10.6
	Std. error	0.08	0.08	0.05	0.05	0.11	0.07	0.06	0.09	0.22	0.14
	Variance	0.09	0.13	0.03	0.04	0.23	0.08	0.05	0.14	0.40	0.31
	Std. dev	0.30	0.36	0.18	0.20	0.48	0.28	0.22	0.38	0.64	0.56

The overall variation for the δ^18^O values of all specimens analysed (A = 4.1‰) ranges from -7.9‰ to -4.0‰. Both M2 and M3 show slightly lower amplitudes of variation (3.6‰ and 3.5‰, respectively), with M2 ranging from -7.6‰ to -4.0‰ and M3 from -7.9‰ to -4.4‰ (Table 4).

Individual δ^18^O values in the sequences represented on each tooth show much lower amplitudes. In M2, the interval of variation ranges from 0.9‰ to 2.8‰ (Mean = 1.6‰), while in M3 it ranges from 1.3‰ to 2.4‰ (Mean = 1.8‰) (Table 4).

The overall variation in δ^13^C values for all specimens analysed (A = 2.6‰) ranges from -12.3‰ to -9.7‰. Individually, both M2 (A = 2.5‰) and M3 (A = 2.0‰) exhibit an amplitude lower than the mean, most notably in the case of M3. In M2, the interval of variation ranges from -12.3‰ to -9.9‰, while in M3 it ranges from -11.7‰ to -9.7‰ (Table 5).

**Fig 3:**
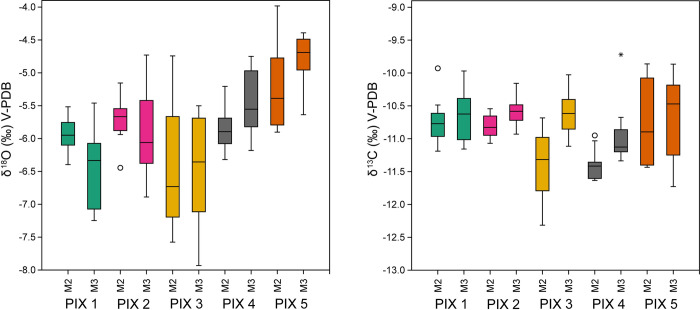
Boxplots showing the range of δ^18^O and δ^13^C isotope values for each tooth of each individual.

δ^13^C values in intra-tooth sequences show much smaller amplitudes. In M2, the interval of variation ranges from 0.5‰ to 1.6‰ (Mean = 1.1), while in M3 it is from 0.8‰ to 1.9‰ (Mean = 1.3) (Table 5 and Fig 3).

Looking at differences between M2 and M3 of the same individual, mean δ^18^O values in M2 are higher than in M3 for PIX1 and PIX2, but lower in PIX3, PIX4 and PIX5. In PIX1, PIX2 and PIX4, the range of values in M2 is lower than in M3 (ca A = 1‰), with PIX3 and PIX5 exhibiting the opposite pattern. Out of the five samples, both teeth of sample PIX3 exhibit the largest amplitude of all the samples (A = 2.5‰) (Fig 3).

In the case of δ^13^C, all M3 exhibit higher mean values than M2. This is especially noteworthy in the case of PIX3 and PIX4, where the One-way ANOVA test shows a significant difference between both means (PIX3, F(1,17) = 20.81, p < 0.001; PIX4, F(1,12) = 7.877, p = 0.016). Notwithstanding, the largest variation can be observed in the M2 of PIX3 and both M2 and M3 of PIX5, with amplitudes ranging between 1.5‰ and 2‰. On the other hand, δ^13^C ranges in PIX2 and PIX 4 are under the 1‰ threshold of variation (Table 5 and Fig 3).

Intra-tooth and intra-specimen δ^18^O sequential values frequently appear as a sinusoidal curve, likely reflecting seasonal change, with the highest values corresponding to the hot season and lower values to the cold season [63].

**Fig 4:**
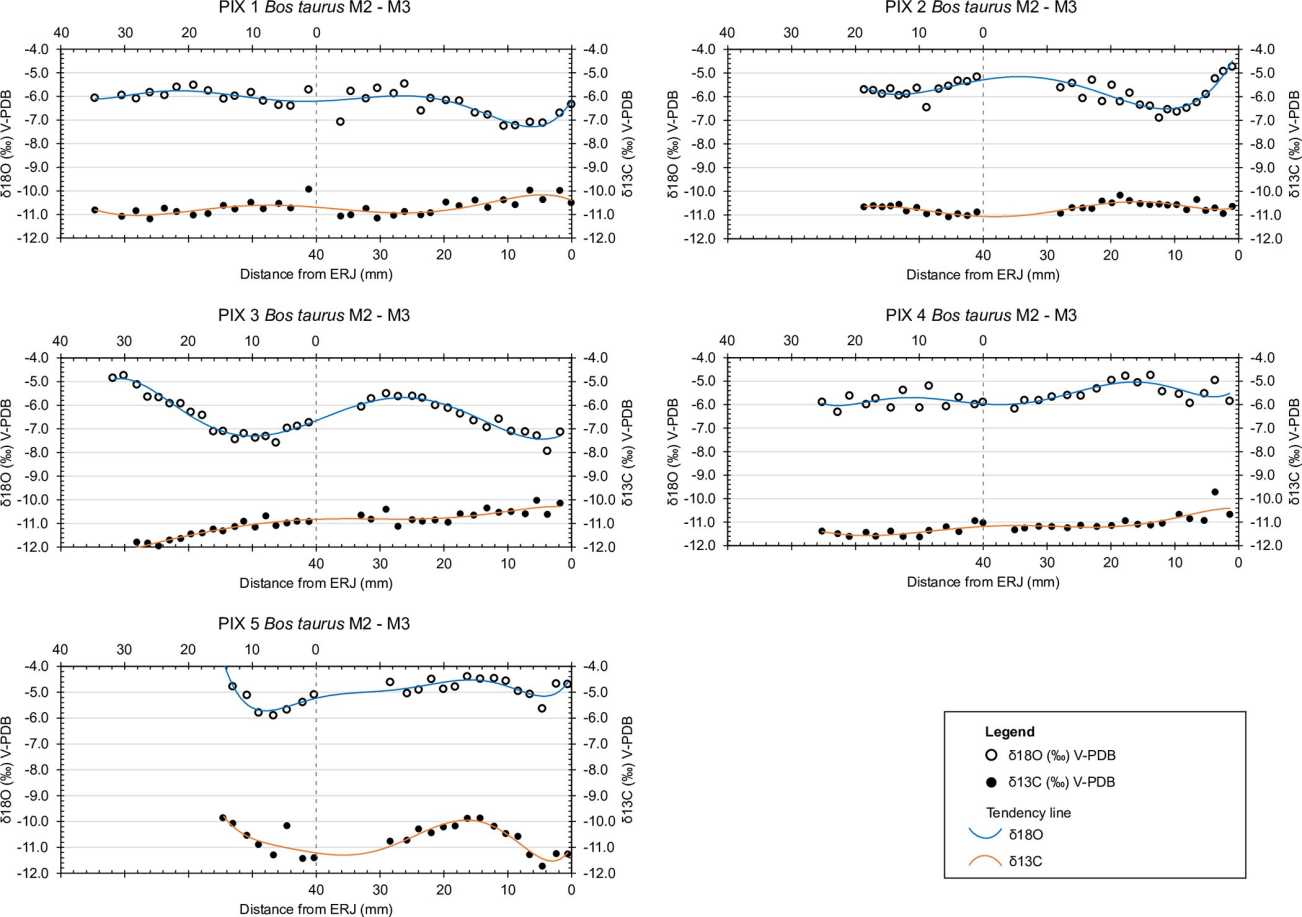
Linked M2 and M3 δ^18^O and δ^13^C isotope curves. Solid lines present the best-fitting curve to the whole sequence prior to the modelling of third molars. The best-fitting curve is calculated as a sixth power polynomial tendency line and assumes a common approximate teeth growth period overlap between M2 and M3 at M2-REJ and M3-40 mm, indicated with a dashed line.

The low degree of variation observed in δ^13^C values results in flattened curves. Even if not following a clear sinusoidal pattern, intra-tooth and intra-specimen δ^13^C and δ^18^O values align with two opposing tendencies, where there is either a positive (PIX5) or negative (PIX1, PIX2, PIX3, PIX4) covariation of the δ^13^C and δ^18^O sequences (Fig 4). In this sense, the higher δ^18^O values observed in the case of both PIX 5 teeth, may be linked to the larger range of δ^13^C. At the same time, it is important to note that the lack of amplitude in the δ^13^C and δ^18^O sequences cannot be attributed to the length of the crown of the analysed teeth. Complete cycles can be observed, thus marking the clear extent of the variation. In a similar way, the largest and smallest amplitudes observed are equally present in long and short crowns.

**Fig 5:**
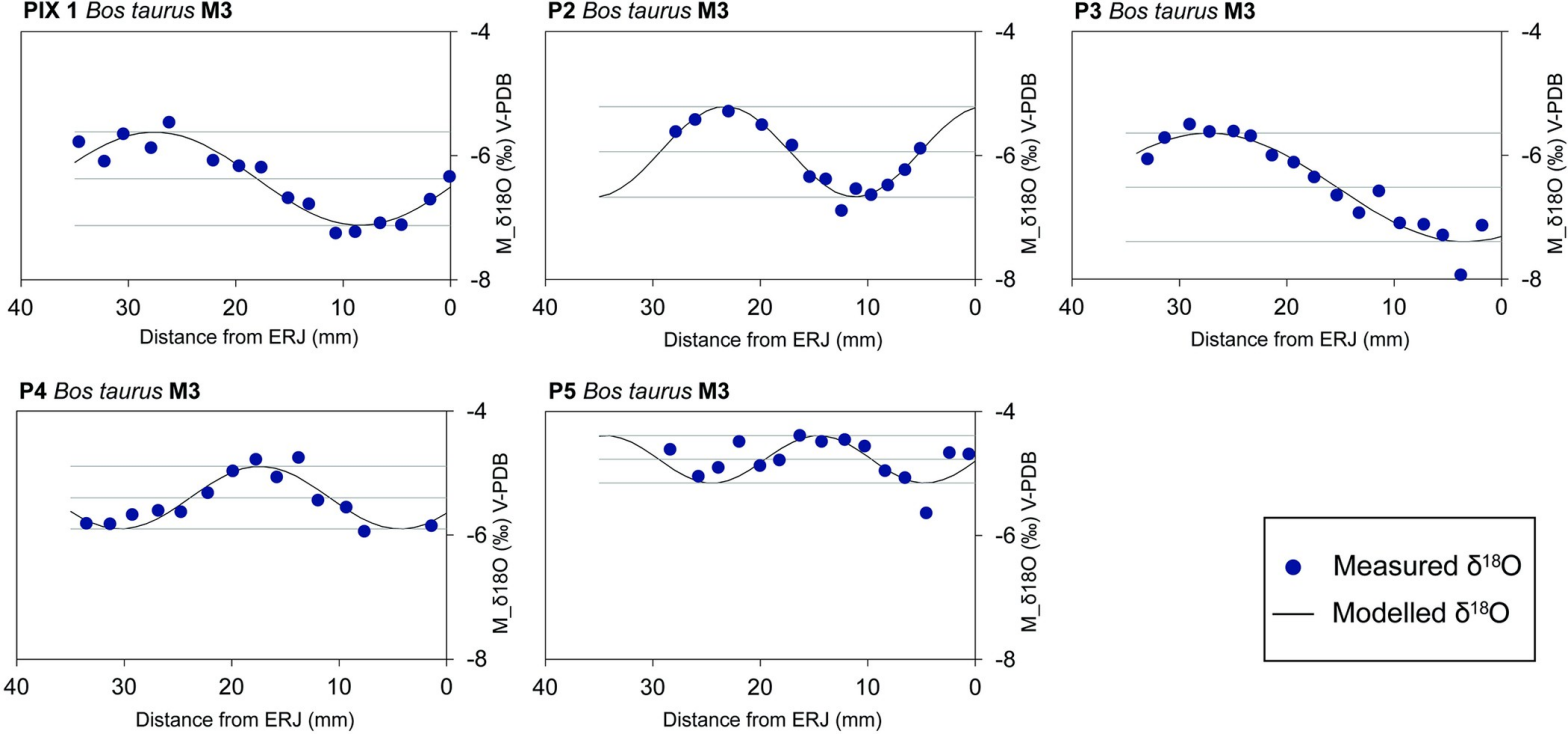
Third molar δ^18^O curve modelling following equation proposed by Balasse and colleagues [64].

Modelling of the birth season was also undertaken on M3 δ^18^O results. The modelled curves and the x_0_*/*X ratios obtained can be observed in Fig 5 and Table 6. Results show that cattle birth occurred during a period of 4.68 months. Further interpretation is only possible in light of comparative data and consequently is reserved for the discussion.

**Table 6:** Description of the factors used in the fitting equation in Fig 4 for each tooth.

	X	A	x0	M	x0/X	MSE	Pearson’s
P1	38.23	0.75	27.57	-6.37	0.72	0.028	0.955
P2	24.21	0.73	23.28	-5.94	0.96	0.011	0.979
P3	47.68	0.88	27.23	-6.52	0.57	0.039	0.959
P4	26.02	0.50	17.37	-5.40	0.67	0.022	0.926
P5	27.16	0.38	21.01	-5.10	0.77	0.014	0.907
				Max	0.96		
				Min	0.57		
				Range	0.39	4.68 months	

Amplitude of the curve (A, in ‰), distance from the enamel-root junction of the maximum value of δ^18^O (x_0_, in mm), value for the annual growth of the tooth (X, in mm) and the mean of the maximum and minimum value (M, in ‰). The x_0_/X ratios obtained vary with the season of birth from 0 to 1 and can be constrained within an annual cycle, providing a measure of the length of the period of births and timing. Mean of the squared error and Pearson´s correlation values are provided as measures of the fitness of the model.

## Discussion

The obtained results provide, for the first time in this region, key insight into cattle feeding practices, reproduction, and herd mobility, crucial aspects to understand Neolithic livestock practices. Additionally, these findings shed light on certain aspects related to the prevailing environmental conditions during this period.

The discussion will first address key climatic considerations which underpin the interpretation of the data. It will then address δ^13^C_coll_ and δ^15^N values to explore overall diet and management prior to slaughter, followed by δ^18^O and δ^13^C_carb_ values to consider seasonal variation in diet, management, mobility and birth season.

### Climate considerations

Climate conditions preceding the Middle Neolithic in the area would have been similar to current climatic conditions but with higher relative humidity [20,75]. At the beginning of the 4^th^ millennium cal BCE, δ^18^O and δ^13^C analyses on sub-fossil oak tree rings provide evidence for limited autumn precipitation [76,77]. Pollen analyses support the expansion of Mediterranean taxa (deciduous broadleaf forests, like oak and beech) in the northeast of the Iberian Peninsula [78]. Deciduous *Quercus* sp., usually associated with *Buxus sempervirens*, is among the most common species documented in the charcoal records of Neolithic sites in the region [79] and has also been documented in Cova de les Pixarelles [49]. Consequently, assessing modern meteoric water δ^18^O values can assist with the interpretation of results, albeit to a limited degree.

Modern seasonal variability in δ^18^O values on a fixed point in the area is around 8‰ [80–82]. Table 7 shows expected modern values for the area at two altitudes, and also the effects on the δ^18^O range of seasonal altitudinal movement between these two points.

**Table 7:** Averaged modern oxygen (δ^18^O) isotope values for meteoric water at two altitudinal levels (900 and 400 m asl) for each month of the year.

	Jan	Feb	Mar	Apr	May	Jun	Jul	Aug	Sept	Oct	Nov	Dec	Max	Min	Range
δ^18^O (‰, V-SMOW) (900 m asl)	-10.6	-10.9	-9.1	-7.6	-5.6	-4	-2	-2.7	-4.4	-7	-9.1	-9.8	-2.4	-10.9	8.5
δ^18^O (‰, V-SMOW) (400 m asl)	-9.6	-9.7	-8.1	-6.6	-4.6	-3	-2	-2.1	-3.6	-6	-8	-8.7	-1.7	-9.7	8
Altitudinal movement	-9.6	-9.7	-8.1	-7.6	-5.6	-4	-2	-2.7	-4.4	-6	-8	-8.7	-2.4	-9.7	7.3

Values are as provided by “The Online Isotopes in Precipitation Calculator, version OIPC3.1. [80,81], based on modern data from the “Global Network of Isotopes in Precipitation data base” [82]. The row “Altitudinal movement” represents a hypothetical value range for potentially consumed meteoric water in a system involving altitudinal movement, resulting in a reduction of the amplitude of δ18O values.

While Cova de les Pixarelles is located at 670 m, the surrounding mountainous plateaus rise to altitudes of 800 m asl to 1000 m asl. The lowest point in the immediate area (c. 400 m), is around the beds of the Balà stream or the river Ter. Considering these data, if the herds moved to the uplands during summer and the lowlands during winter, the δ^18^O curve would show a certain degree of flattening (0.7‰–1.2‰, less amplitude) as a result of the seasonal consumption of water at different altitudes, even within limited altitudinal differences and small geographical displacement. It is important to note, however, that these are averaged modern values. Specific years could potentially have a greater effect, and the differences in humidity during the Middle Neolithic could also have an impact on variability. This aligns with the results obtained in previous studies conducted on sheep and goats [83–90], including a study of a modern transhumant herd moving between the Ebro valley and the central Pyrenees. Furthermore, other studies in the area [91,92] demonstrate that altitudinal mobility is bound to produce a flattening of the δ^18^O curves but will also show a negative covariation of δ^18^O and δ^13^C values along crown sequences. These studies demonstrate that δ^18^O measurements in tooth enamel bioapatite accurately capture the seasonal variation observed in meteoric water across the study areas. These δ^18^O variations were consistent regardless of the seasonal vertical mobility of the sheep and other potential influencing factors. In contrast, the δ^13^C values in plants showed a clear altitudinal gradient, becoming progressively more depleted at higher elevations, which reflects differences in precipitation patterns and vegetation availability across the sites. The environment of the Collsacabra natural region is expected to exhibit the same patterns of isotopic variability, as they are all included in the Cfb Köppen-Geiger Climate classification [93]. The available data for the nearby regions of La Garrotxa and Girona also suggest a landscape dominated by deciduous forests, with a particular emphasis on oaks [94]. While human impact during the Early Neolithic in these two regions caused a regression in favour of grasses [94], the presence of oak remains in Cova de les Pixarelles [49], can be associated to the small expansion of holm oaks observed in these regions during the transition to the late Holocene [94].

### Diet

Overall, the δ^13^C and δ^15^N values of the analysed cattle samples from Cova de les Pixarelles show limited variability. Cattle likely grazed in fairly similar open environments that remained largely unaffected by human activity. Cattle grazing would probably occur in a mountainous grassland environment where wild legumes were abundant. To date, no other plant remains have been found in the cave, apart from fragments of burnt oak branches and acorns. Although it can be assumed that agricultural activity was part of Neolithic life, no domestic plants have yet been identified in the site that would have contributed to cattle feeding.

The few relatively low δ^13^C values could indicate that some cattle might have been more reliant on or have access to food sources presenting more depleted δ^13^C values. Considering the landscape characteristics of the site, pasturing in forested areas or forest limit, might have produced this kind of effect [95]. By contrast, the aforementioned samples from La Draga that stand out for their higher δ^13^C values, a phenomenon that had previously been attributed to supplementing the animals’ diet with stored agricultural by-products [74,96] like cereal chaff or grain [97]. Compared to the available carbon and nitrogen isotope data for Neolithic cattle in the northeastern Iberian Peninsula, the results for Cova de les Pixarelles show in average the most depleted δ^13^C (m = -20.6‰) and δ^15^N values (m = 4.4‰). Considering the 3–5‰ diet-consumer shift [98–100], it is possible that leguminous plants had a significant role in their diet. This is in line with hypotheses supporting a lower value for plants that fix nitrogen from the atmosphere compared to the ones that fix it from the soil [101], normally yielding values around 0‰ [102]. The Iberian Peninsula is nowadays very rich in wild leguminous plants,which are an essential part of grasslands and pastures. Many of them are commonly used for livestock grazing and can be seasonally harvested (e.g. the genus Trifolium (clover) or *Medicago sativa* (alfalfa), nowadays among the cultivated fodder plants) [103]. In that context, Tornero and colleagues [91], also reported an abundance of wild leguminous plants in their study on sheep vertical mobility between the Ebro Valley and the Pyrenees. The low δ^15^N values also suggest that the exploited pastures had not been improved through anthropogenic activity, such as manuring. This evidence contrasts with La Draga and Reina Amàlia, where different grazing and feeding strategies seem to have been implemented [74,96]. In this sense, it is important to note that both sites are examples of consolidated farming communities [104].

#### Seasonal variation in diet

In the case of δ^13^C_coll_, the diet-consumer shift for herbivores usually represents an enrichment of 5‰ [105,106]. In the case of ruminants, bioapatite represents a mean enrichment of 14.1‰ [107] from food source to consumer. Given this, both δ^1^^3^C_coll_ and δ^1^^3^C_carb_ align with the δ^1^^3^C values of the food sources (-25.8‰ and -23.8‰), falling within the pre-industrial CO₂-corrected global range of δ^1^^3^C variation for C3 plants from open, temperate environments (−31.5‰ to −23‰) [108,109]. The low intra-tooth variation of δ^13^C_carb_ values of Cova de les Pixarelles closely resembles the LBK cattle from Ludwinowo [3]. In this particular study, the flat δ^13^C curves are argued to be the result of “little seasonal variation in pasture and fodder sources”. It is also argued that leafy fodder would not have been provided to these animals, as δ^13^C would decrease with the decrease of δ^18^O values [3,110].

The flattening of the δ^13^C curves aligns well with the lack of variation observed in the bulk collagen samples and is probably largely explained by the altitudinal gradient in water availability and the reduction in δ^13^C values associated with changes in plant diversity and functional variation with increasing altitude [92,111]. If there are other environmental conditions affecting the δ^13^C values, they are either masked by altitudinal variation or affects animals in a similar way throughout the year. In the Iberian Peninsula, plant δ^13^C can vary up to 4‰ [112–114] during the seasonal cycle, thus constraining the values we can potentially record on animal tissues. La Draga could serve as an example of this. Considering that the herds probably lived at the site all year round [74,97], the sequential δ^13^C values reflect a range of less than a 2‰ variation [74], not very far from the average values presented by sheep and goats at the same site [74]. At Cova de les Pixarelles, δ^13^C_coll_ values for the mandibles and teeth analysed in this study are representative of a C_3_ diet and have a limited overall range of only 1.5‰. It is also important to highlight that combining data from dentine (representing short-term early life signals) and mandibular bone (representing a longer-term average) still gives a very limited range. However, it should be noted that adding the baseline data from Pixarelles (C. de les Pixarelles B) (Tables 2 and 3) significantly increases the amplitude of variation in the observed δ^13^C values. The different time scales of long bone remodelling may explain this difference. Bioapatite δ^13^C values show little seasonal variation. With the exception of PIX5, which shows a more usual seasonal variation of about 2‰, the curves of PIX 1 to 4 appear fairly flat. The length of the crown available for M2’s largely precludes the possibility of observing a dietary shift with the start of rumination after birth, if there was one.

All three sites reflect animal management strategies well integrated with the economic practices of each community, making the best out of the environmental conditions that surround the sites. On one hand, in La Draga and Reina Amàlia the human communities directly modified and adapted their environment to facilitate farming practices. On the other hand, in Cova de les Pixarelles they adopted tailored management practices that allowed them to provide the animals with the best quality pastures while at the same time controlling their nutrition, reproduction and mobility. The lack of direct evidence supporting agricultural practices in Cova de les Pixarelles might be one of the reasons that allowed or encouraged the development of this approach to animal management. Biomechanical and palaeopathological analyses suggest that very few of these animals could have been used as working animals [40,59,115], which does not necessarily entail agriculture as part of the equation [9]. All these factors do not necessarily argue against the practice of agriculture by the inhabitants of the cave. Coordinating a domestic herd and a camp herd within an Open-Range Cattle Grazing system could be a functional approach to ease the agricultural tasks and the direct dedication to herd maintenance. The observed characteristics highlight the preeminent role of husbandry in the activities developed in the cave and the likely optimal animal management strategy to cover food needs.

### Mobility

Based on the abundance of C_3_ and C_4_ plants at different altitudes in Kenya [83] noted that variations in δ^13^C values could be a good indicator for altitudinal mobility. Further studies with sheep and goats [86,88,89,91,92] have shown that monitoring both δ^13^C and δ^18^O can provide a much clearer picture of this type of mobility. In this sense, the inverse co-variation of δ^18^O and δ^13^C (having the δ^13^C maximum values match with the δ^18^O minimums and *vice versa*), is usually consistent with a pattern of seasonal altitudinal movement. As observed in modern values [91] (See “Climate considerations” in this article) seasonal altitudinal movement in the area would result in a slight flattening of the meteoric water δ^18^O curve.

The sequential δ^18^O and δ^13^C results from PIX1 to 4 are probably the result of this scenario. This tendency seems clear in PIX1 to 3, already from the cycle represented in the M2. However, it cannot be observed on PIX4´s M2, only in the M3 sequence, which raises the question of the possible practice of the vertical mobility strategy later in the animal’s development. PIX5 presents a different case, where both δ^13^C and δ^18^O follow the natural trend of seasonal variation. This is likely the result of free-range pasturing, without altitudinal mobility, but rather grazing on plants on a fixed area.

A specimen from Džuljunica [1] shows a similarly flat δ^13^C pattern with a more pronounced fluctuation in δ^18^O levels, potentially due to harsher winters. The authors interpret this pattern as evidence of limited mobility and occasional winter foddering with plants with high δ^13^C values. However, unlike our findings, their δ^13^C data lacks observable seasonal variation, and the annual fluctuation is far less linear than what we observe at Cova de les Pixarelles. We agree that their proposed interpretation is the most reasonable, but also does not conflict with our hypothesis. As Makarewicz [86] noted, distinguishing between winter foddering and vertical mobility strategies remains challenging.

The six cattle from Tana del Barletta (Ligurian Prealps) [116] provide a similar context and results to those observed at Cova de les Pixarelles. The δ^18^O curves show a particularly narrow amplitude of variation (from 0.9 to 2.9‰, m = 1.6), a characteristic that the authors link to vertical movement between the site and a potential lowland settlement [117,118].

When considering the possibility of winter foddering strategies as an alternative interpretation to mobility, we must consider how these are reflected in the δ^13^C curve. In the case of La Draga [74] supplementary feeding appears as a peak curve that visibly offsets the normal correlation of the δ^18^O and δ^13^C sequence. While this would be representative of a punctuated input of food, rather than sustained foddering, it also illustrates to what degree supplementary feeding can modify δ^13^C curves. Similarly, the sheep from the Orkney Islands feeding on seaweed also show a large enrichment of ^13^C values [119,120]. In the case of Cova de les Pixarelles, no such alteration can be observed in any of the analysed animals, whether mobile or not. At the same time, winter foddering on collected forestry would provide depleted ^13^C values because of the canopy effect [95]. This would oppose the tendency observed in the mobile animals from Cova de les Pixarelles which show slightly higher ^13^C values during the cold season.

Not many studies have addressed cattle mobility through sequential δ^18^O and δ^13^C, but evidence from Cova de les Pixarelles, match well with the results from Tana del Barletta [116], and mobility patterns observed in mobile sheep and goat herds [91,92] in similar environments. The existence of transhumance practices during the Neolithic have been long proposed and discussed throughout the Mediterranean basin [121]. However, it is frequently stressed that full scale, long distance transhumant practices have only been clearly identified well into the Bronze age [18,121]. At the same time, recent studies suggest that the various forms of small-scale transhumance such as transterminance [20,25,26,30], with their own range of general and more specific or regional terms used to address these phenomena [122], probably started during the Early Neolithic [20,90,123,124].

In Cova de les Pixarelles, four of the animals analysed show a vertical mobility pattern, while a fifth would remain in similar conditions throughout the year. These two patterns could reflect complementary strategies in herd management (e.g. keeping male and female separate), which could be consistent with Tefera’s ecological division of herds.

### A timely birth

**Fig 6:**
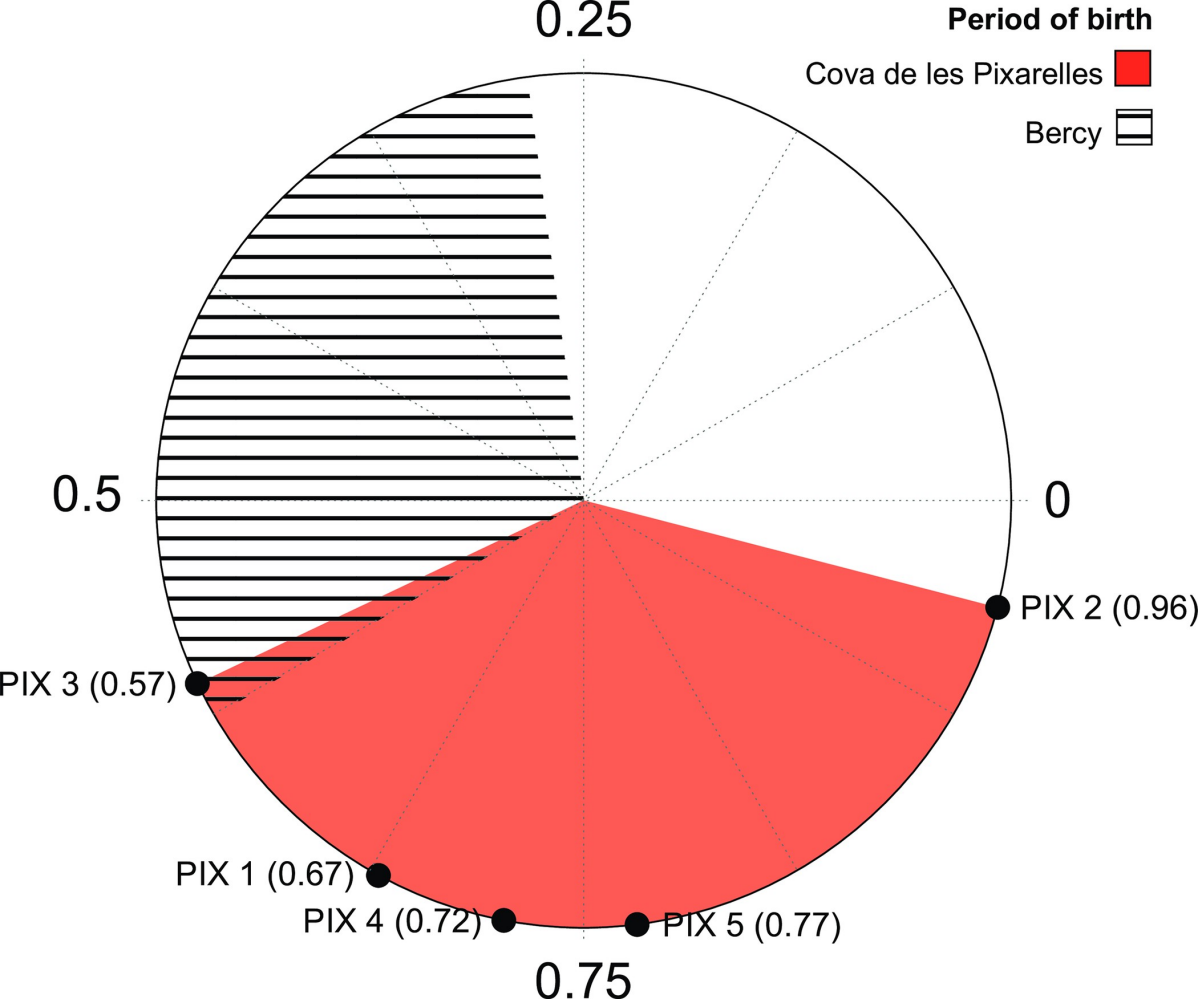
Circular representation of Cova de les Pixarelles cattle season of birth based on δ^18^O curve modelling following Balasse *et al* 2012. Reference for the main season of birth for cattle (spring) is the Bercy herd [9]. Subdivision of the circle in quarters allows for an approximate representation of seasons.

The application of the model proposed by Balasse and colleagues [64] to the δ^18^O datasets of Cova de les Pixarelles indicate that cattle birth occurred within a period of 4.68 months (Figs 5 and 6, and Table 6).

While cattle and aurochs can reproduce all year round, available data suggest that their birthing season is largely limited by resource availability and is thus usually restricted to a 3-month period in spring [9]. In domestic animals, there appears to be a slight delay in the season of birth, as well as a slightly wider birthing period. While it is not uncommon for births to occur later in the year in both aurochs and cattle, they are usually not later than mid-summer. In cattle, the occurrence of births outside of the optimal period, and especially after mid-summer, likely involve human manipulation [9].

Looking at the available data on cattle’s season of birth in European contexts [1,9], the most notable feature of Pixarelles is that the analysed specimens were mainly born outside the main period of births for aurochs. Only PIX3 falls within the very end of the extended birth period for domestic cattle. PIX1, 4 and 5 are born later, but within a month, and PIX2 stands alone at the end of the fourth quarter (Fig 6). A similar disposition can still be observed if we consider a -0.21 average correction to the x_0_*/*X values from the hypoconid and protoconid as observed for the LBK cattle from Chotěbudice and Černý Vůl [69]. While we cannot pinpoint the exact season of birth as with sheep, the available information indicates that the main period of cattle birthing occurs during the spring months [1,8,9,65–69]. After modelling δ^18^O curves and dividing the representation in 12 equal portions, emulating the months of the year, spring loosely matches with the three portions corresponding with the second quarter, thus allowing some indication of season. All things considered, the births in Pixarelles would seem to mainly occur during summer, with one individual in late autumn. It is important to note that even with the extensive work dedicated to caprines birth seasonality, late summer/autumn births are still difficult to pinpoint for these species [125]. Although new referential works are addressing the issue in caprines [91,125–127], substantial error is still expected regarding births in this part of the year. Given the lack of similar studies for cattle, a similar outcome needs to be considered. This, however, does not contravene that the birth season of the analysed cattle falls out of the expected seasonal range [128,129]. Even in the modern feral populations of cattle in the Doñana National Park, south of the Iberian Peninsula, births largely occur during spring [130,131].

Considering this, such a delay in the birth season of cattle is certain to represent human manipulation. In this sense, considering the hypothesis for seasonal altitudinal movement, it seems likely that cattle births were timed to occur during the first months spent in the summer pastures. Reports of early XXth century cattle and sheep births in transhumance and vertical mobility regimes in the Iberian Peninsula are opposite to this dynamic [132,133]. Animal births are targeted to occur from December to February and March at the latest [133]. But at the same time, herders consistently complained about the costs of the fodder needed to cope with this strategy and the need to link the transhumance practice with agriculture [132]. While we should consider the differences between the Neolithic and modern productive goals and economic system, the size of herds and the accumulated impact on natural resources, these modern cases exemplify an essential need for herd maintenance: the provision of high-quality food during and after the birth season. At Cova de les Pixarelles, cattle births seem to occur after the seasonal movement of the herds to fresh pastures in spring or early summer, and their return to their home pastures by autumn, providing cows and calves with the essential high-quality grazing vegetation. While the exploitation strategy at Cova de les Pixarelles does not seem primarily focused on milk production, it is a factor to consider. Such a seasonal approach would make milk most available in summer, and the late autumn birth would contribute to milk availability into the winter. Such a timing strategy, where birth seasons align with human needs, has been identified in other European sites [9], and the cattle at Cova de les Pixarelles appear to follow a similar pattern. The overlap between birth timing and the milk production period suggests that milk management may have been a contributing factor to the observed birth patterns. The distribution of births could, however, primarily reflect herd management choices aimed at maximizing herd survival and productivity, rather than being solely driven by the need for milk exploitation.

In this sense, Tefera´s [32] concepts on the ecological division of the herds can provide a straightforward explanation. Reproductive specimens, can be isolated as part of the “domestic herd”, kept safe close to the settlement while the remaining “camp herd” is brought to fresh pastures. This creates a situation where the breeding season is, to a degree, dependent on both herds being kept together. In recent transhumant cattle herds from the Iberian Peninsula, the return to winter pastures can occur up to October [132]. Adding to this the gestation period of cows [134], we can match the breeding season for the cattle of Cova de les Pixarelles to the return to the winter pastures and the reunion of both herds, which can explain the birth season shift documented.

### Managing the cattle herds

The results discussed so far draw a complex picture of the cattle management practices implemented in Cova de les Pixarelles. Based on the isotopic evidence, the cattle from Cova de les Pixarelles had an indirectly managed diet, in the sense that no supplementary feed was provided, but their pasture areas were selected, either by moving the animals seasonally or by not allowing them to. Four of the animals exhibit variations in the δ^18^O and δ^13^C sequences that align with a seasonal altitudinal movement. At the same time, a fifth one would have not been included in these seasonal movements and likely stayed in the same environment all year round. Regarding their season of birth, most of the cattle analysed from Cova de les Pixarelles are born out-of-season or late on the expected season of birth for this species. Out-of-season births in domestic cattle are not impossible or uncommon at all [9]. Considering that its wild ancestor´s season of birth was during spring or very early summer, as it is the most usual too for domestic cattle, out-of-season birth certainly points in the specific direction of human intervention. The purpose and extent of this practice is something that needs to be further investigated.

Evidence for cattle from Cova de les Pixarelles stemming from the archaeozoological, palaeopathological and biomechanical studies can complement the observations made so far. Cattle were certainly killed for their meat, and likely other post-mortem products [40]. Processing cut marks and fresh bone fractures argue in that regard. Palaeopathological observations indicate a generally healthy herd, but with some healed or healing bone trauma, and a low pathological index (IPI = 0.149, after Bartosiewicz and colleagues) [135], or lower, following Thomas and colleagues’ [136] considerations) for the available phalanges [58,59], commonly used as markers of the use of cattle for traction [137]. The biomechanical studies of these cattle bone phalanges linked their observed higher mechanical stress to their habitat [40,115]. This is because the mechanical loading observed in the phalanges generally favoured the forelimb, as opposed to the “anteriorization” of posterior phalanges that occurs on labour cattle [39]. At the same time, the phalanges from Cova de les Pixarelles generally exhibited a good adaptation to multidirectional strains, and a greater incidence of lateral strains, which defined the lack of restrictions to their mobility but also their need to counter uneven terrain. However, they also noted that very few of these animals could have performed more straining, maybe load-bearing, work-related activities [40,115], adding perspective to the palaeopathological observations.

The evidence collected so far draws a new picture of the herding practices during the Middle Neolithic of the Iberian Peninsula, based on the ecological division of the herds [32] and an Open-range cattle grazing model [31]. The Open-range cattle grazing model allows cattle to roam freely, finding food for themselves on broad landscapes, which requires less work investment from the herders. This seems to be the general approach to cattle management for Cova de les Pixarelles. The geography of the mountains in the area would easily allow it, and the isotopic results regarding diet, supporting feeding in an open environment without traces of anthropogenic modifications, also lean to this hypothesis. However, having identified different mobility regimes supports the implementation of complementary regimes. The ecological division of the herds explains why some of the animals were moving seasonally (“camp herds”) while others were not (“domestic herds”). The separation of the herd relieves pressure on the pastures of the “domestic herds”, provides fresher pastures for the “camp herds”, and is effectively used to separate reproductive specimens, regulating or modifying the reproduction rhythms of the herd. In this sense, both groups would still be allowed to roam freely, but the “domestic herd”, which ensures the reproduction of the herd, would be kept within grasp. The combination of these two concepts explains the characteristics observed regarding alimentation, mobility and reproduction, allowing minimal human intervention, and thus a reduction in workload, and maintaining a certain degree of control over the herd.

These data are in line with the general interpretation of the Neolithic settlement dynamics of the area and the specific use of the Pixarelles cave [57]. The cave is located at a point of contact between different morphostructural units. The river valley forms a natural corridor between the inland plains and the pre-coastal and coastal areas. There are few caves that show human occupation in this period. Most settlements are open-air. The caves were used as settlements with a specific purpose, such as burials [57]. The existence of other burial caves and a group of megalithic monuments a short distance from the Pixarelles cave, on the upper plateaus [57], shows a complex dynamic of interactive use of the territory during the 5^th^ and early 4^th^ millennium BCE in this area of rugged landscape. A settlement model that combines a regime of economic or social complementarity between the settlements of the higher areas with those of the inner plains is the most probable scenario. The interdisciplinary study of the open-air settlements of this chronology has shown that they had a stable economy based on cereal farming and livestock rearing centred on the management and exploitation of sheep and cattle, with a minor presence of pigs. In this context, and as this study has demonstrated, cattle farming would be partly extensive, taking advantage of seasonal grazing. Regarding Cova de les Pixarelles, all the remains studied correspond to a single level of intensely anthropized occupation, which seems to be structured around a hearth [49]. We must assume that the purpose of the fireplace was to provide light and heat, without excluding its culinary use. A working area was created around the hearth. Taphonomy analysis of the faunal remains has documented the presence of fresh fractures, and few traces associated with food consumption [40]. Of the 456 faunal remains analysed, only 8 show signs of thermal alteration. Therefore, these remains are mainly from the processing or preservation of meat, with little evidence of direct consumption [40]. Bone refitting and anatomical connections indicate that it is a collection with a high level of integrity, probably formed over a short period of time [40]. By combining the documented territorial management of the bovids with the specific practices observed in this cave, the evidence suggests an integrated approach to land use. This approach likely involved a network of interconnected settlements spanning both the plains and higher elevations, with the caves serving a distinct purpose. In the case of Cova de les Pixarelles, probably related to the acquisition and long-term preservation of food supplies. In this context, the cattle would probably be subject to communal management.

## Conclusions

Due to its abundance and exploitation patterns which focused on meat, the accumulation of cattle in Cova de les Pixarelles is probably linked to its importance as a source of meat and the efforts of human communities to preserve and store it. The cattle from Cova de les Pixarelles were reared according to two strategies, depending on their productive and reproductive function: one that involved seasonal mobility, changing from lowland pasture areas to the mountain plateau, and one that involved moving between pastures in the same area. Through these complementary strategies, the availability of food was ensured year-round. Otherwise, no supplementary feeding in their diet could be detected. The concepts of Open-range cattle grazing (free-range grazing) and the ecological division of herds explain well the data available for Cova de les Pixarelles. Managing separated herds, whether on the basis of age and sex or productive function, enables a degree of control over reproduction. In the case of Cova de les Pixarelles this resulted in out-of-season births: during summer, matching the access to new pastures, and in late autumn, potentially allowing for winter access to milk.

In conclusion, the cattle from Cova de les Pixarelles support that Middle Neolithic human communities made wide use of the available ecological resources by implementing complementary management strategies that were developed based on the productive and reproductive goals of their cattle herds. These strategies used allowed them for yearly planning of the birth season and ensured adequate pastures year-round through the mobility of the animals.

## Supporting information

S1 Fig Age profile of cattle from Cova de les Pixarelles based on tooth wear [138,139]. (TIF)

S2 Fig Percentage of unfused and fused skeletal elements of *Bos taurus* remains from Cova de les Pixarelles by fusion stages. (TIF)

S3 Fig Flexible mixture analysis of linear breadth measurements [140] of long bones evaluating potential sexual dimorphism within the assemblage. (A) Greatest breadth of the distal end (Bd) of the Metacarpal. (B) Greatest breadth of the distal end (Bd) of the Metatarsal. (C) Greatest breadth of the proximal end (BP) of the Radius.
(TIF)

S1 Table Skeletal representation of *Bos taurus* NISP of every major bone (B) identified within the assemblage of Cova de les Pixarelles and NISP within every Anatomical Group (AG). (DOCX)

S2 Table Carbon and Nitrogen data prepared for this paper from the cattle recovered in the sites of Cova de les Pixarelles, Reina Amàlia and la Draga. The table displays all the samples with collagen analysed. Samples with C:N ratios over 3.6 were discarded and were not incorporated in the baseline.
(DOCX)

S3 Table Cattle teeth enamel sequential carbon and oxygen results from Cova de les Pixarelles. Distance of the samples from the Enamel-Root Junction (ERJ dist.) is expressed in millimetres. δ13C and δ18O values are expressed in ‰ and corrected using the Vienna Peedee Belemnite (VPDB) standard.
(DOCX)
