# Correction: Cattle on the rocks: Understanding cattle mobility, diet, and seasonality in the Iberian Peninsula: The Middle Neolithic site of Cova de les Pixarelles (Tavertet, Osona)

**DOI:** 10.1371/journal.pone.0328468

**Published:** 2025-07-15

**Authors:** Roger Alcàntara Fors, Richard Madgwick, Laura C. Viñas-Caron, Alexandra J. Nederbragt, Maria Saña Seguí

There are errors in the Funding statement. The correct Funding statement is as follows: R.A. is supported by the Margarita Salas Postdoctoral Grant (730263), funded by the Ministerio de Universidades and the European Union-NextGenerationEU. M.S. acknowledges financial support from the Institució Catalana de Recerca i Estudis Avançats (ICREA) through the ICREA Academia program (2021–2). This research was funded by the ANIMAL FARM (PID2020–115715GB-I00/AEI/10.13039/501100011033) and FRACTURES (PID2023-00X152137IV0) projects, both financed by the Ministerio de Ciencia, Innovación y Universidades, with M.S. as PI, and by the project Convocatoria de analíticas 2022–2023: Estudios paleomoleculares de restos de fauna de yacimientos neolíticos del Noreste peninsular: dilucidando la alimentación, reproducción y movilidad de los primeros rebaños domésticos (Palarq 2022/23–82), funded by the Palarq Foundation and also led by M.S. The funders had no role in study design, data collection and analysis, decision to publish, or preparation of the manuscript.

The reference citation in Fig 2 caption is incorrect. Please see the correct Fig 2 caption here.

**Fig 2:**
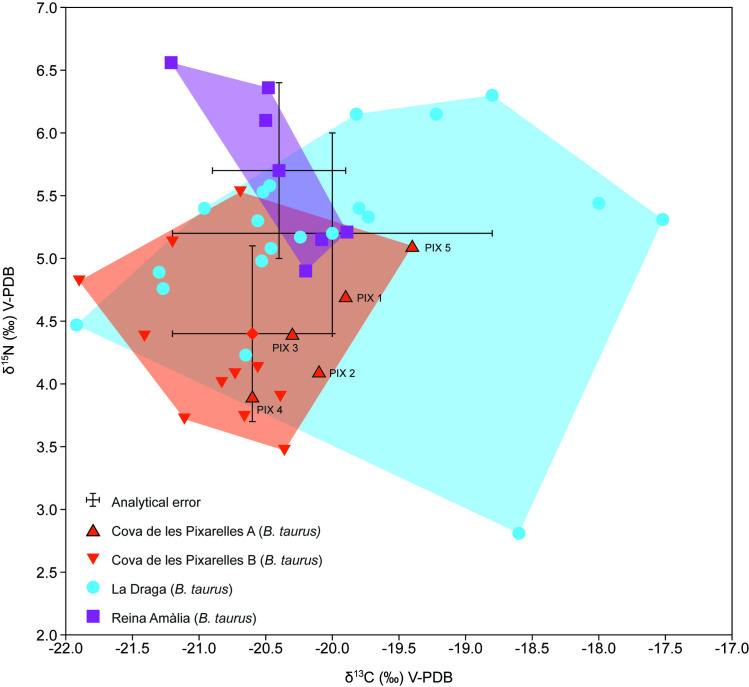
Scatter plot with the. δ13C and δ15N values of *Bos taurus* from Cova de les Pixarelles, LaDraga and Reina Amàlia.

The dataset includes the results of *Bos taurus* mandible samples from Cova de les Pixarelles (n = 5) and *Bos taurus* baseline values from the sites of Cova de les Pixarelles (n = 11), La Draga (n = 19) and Reina Amàlia (n = 4) prepared for this study and previously published values [74] from La Draga (n = 2) and Reina Amàlia (n = 2). The polygons represent the bivariate range for each dataset.
