# Supplementary figures and images for: Cattle on the rocks: Understanding cattle mobility, diet, and seasonality in the Iberian Peninsula. The Middle Neolithic site of Cova de les Pixarelles (Tavertet, Osona)
Source: PLoS One. 2025 Jan 27;20(1):e0317723. doi: 10.1371/journal.pone.0317723 (PMC11772053; doi:10.1371/journal.pone.0317723)

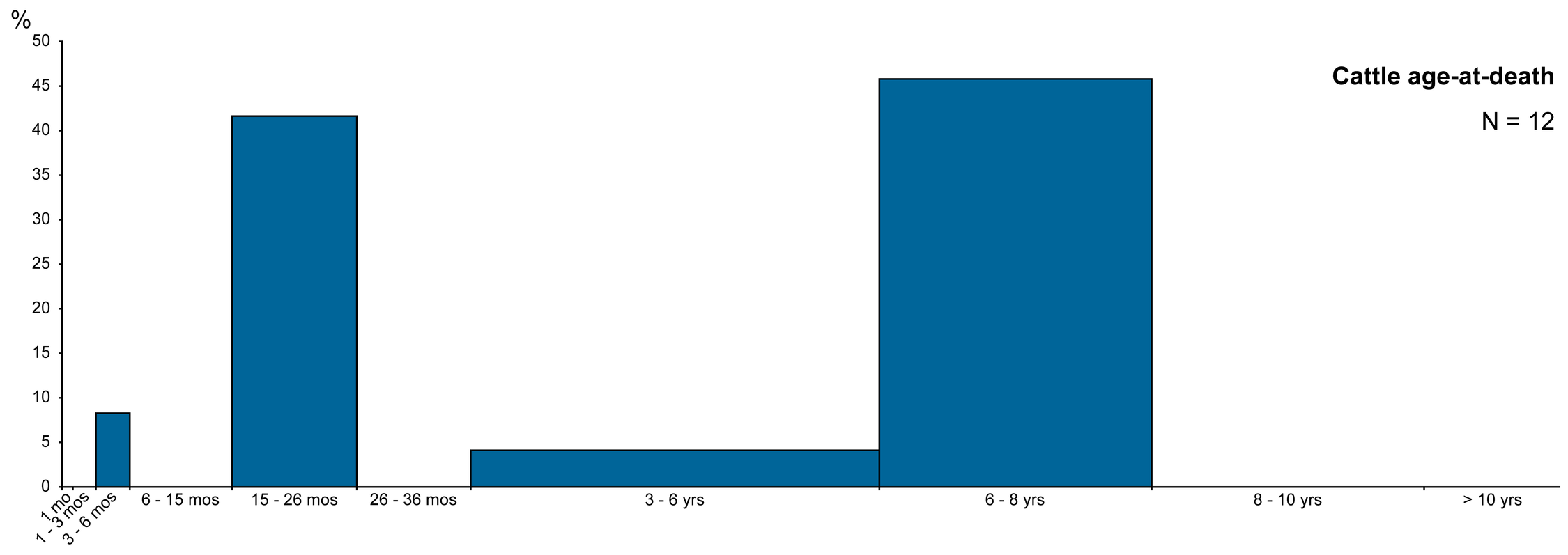

Supplement: S1 Fig — (TIF) [file pone.0317723.s001.tif]

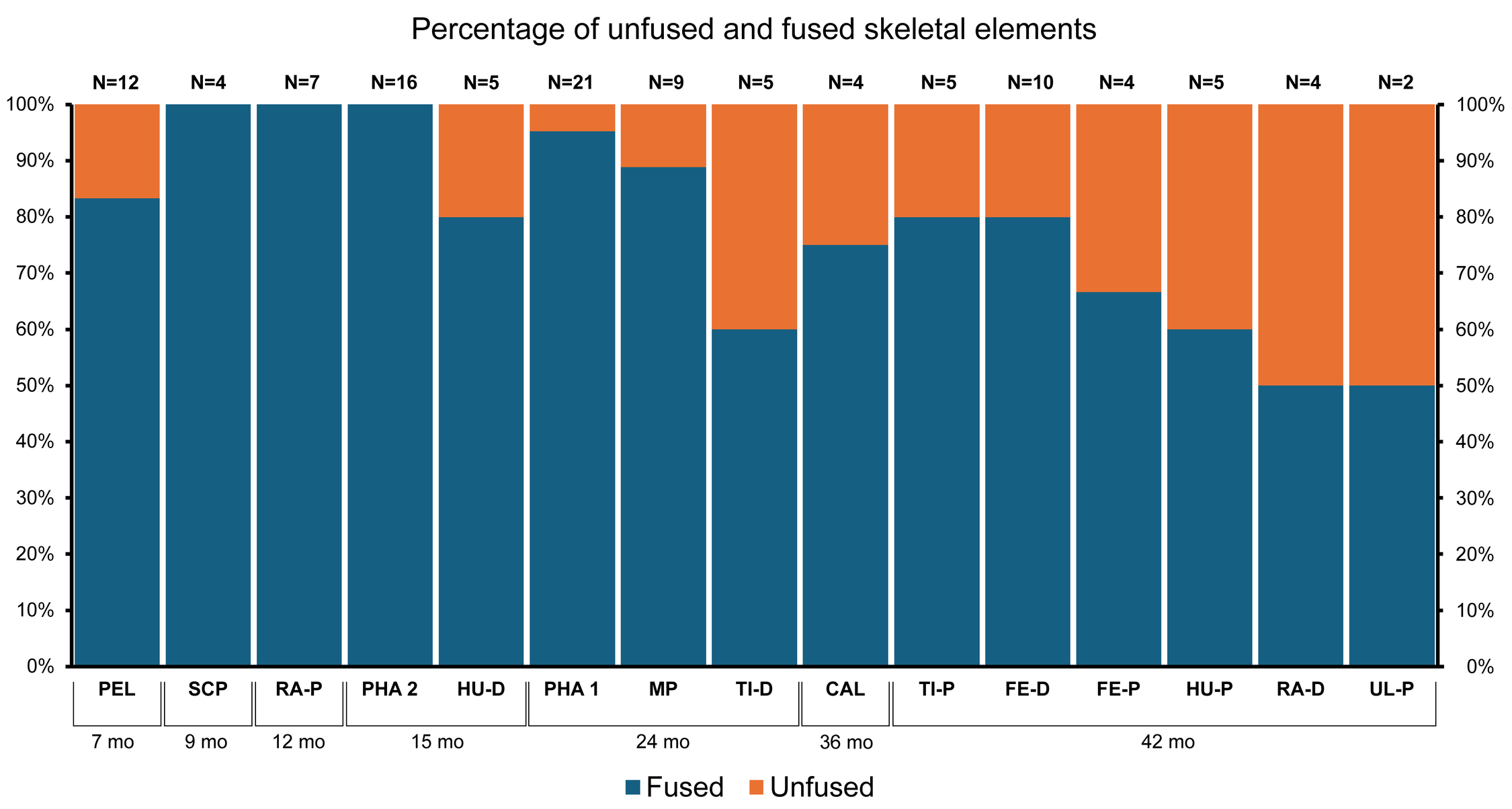

Supplement: S2 Fig — (TIF) [file pone.0317723.s002.tif]

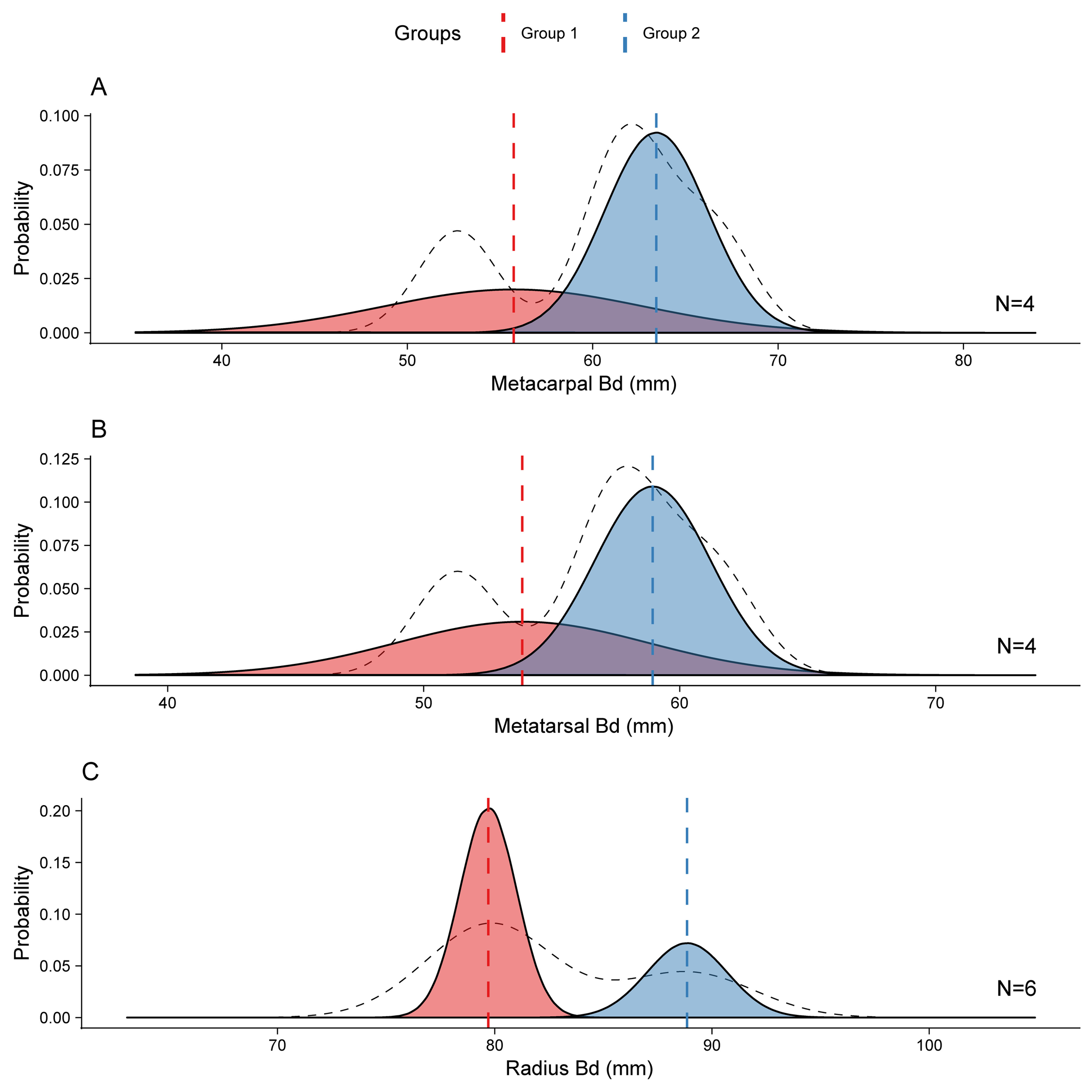

Supplement: S3 Fig — (A) Greatest breadth of the distal end (Bd) of the Metacarpal. (B) Greatest breadth of the distal end (Bd) of the Metatarsal. (C) Greatest breadth of the proximal end (BP) of the Radius. (TIF) [file pone.0317723.s003.tif]
