# Supplementary material for: Cattle on the rocks: Understanding cattle mobility, diet, and seasonality in the Iberian Peninsula. The Middle Neolithic site of Cova de les Pixarelles (Tavertet, Osona)
Source: PLoS One. 2025 Jan 27;20(1):e0317723. doi: 10.1371/journal.pone.0317723 (PMC11772053; doi:10.1371/journal.pone.0317723)
Supplement: S1 Table — (DOCX) [file pone.0317723.s004.docx]

**Cattle on the rocks: Understanding cattle mobility, diet, and seasonality in the Iberian Peninsula. The Middle Neolithic site of Cova de les Pixarelles (Tavertet, Osona)**

**Roger Alcàntara Fors^1,2*^, Richard Madgwick^1*^, Laura C. Viñas-Caron ^2,3^, Alexandra J. Nederbragt^4^, Maria Saña Seguí^1*^**

**Supporting information**

**S1 Table. Skeletal representation of *Bos taurus* NISP of every major bone (B) identified within the assemblage of Cova de les Pixarelles and NISP within every Anatomical Group (AG)**

| **Anatomical Groups** | **Bone** | **NISP (B)** | **NISP (AG)** |
| --- | --- | --- | --- |
| **SKULL** | Skull | 11 | 54 |
|  | Horn | 5 |  |
|  | Mandible | 26 |  |
|  | Maxilla | 12 |  |
| **TORAX** | Atlas | 4 | 135 |
|  | Axis | 1 |  |
|  | Ribs | 96 |  |
|  | Caudal vertebrae | 2 |  |
|  | Cervical Vertebrae | 9 |  |
|  | Lumbar Vertebrae | 13 |  |
|  | Toracic Vertebrae | 7 |  |
|  | Sacrum | 3 |  |
| **DISTAL LIMBS** | Carpal bones | 4 | 42 |
|  | Tarsal bones | 3 |  |
|  | Calcaneus | 1 |  |
|  | Metacarpal | 4 |  |
|  | Metapodial | 1 |  |
|  | Metatarsal | 5 |  |
|  | First phalange | 7 |  |
|  | Second phalange | 4 |  |
|  | Third phalange | 11 |  |
|  | Talus | 2 |  |
| **PROXIMAL FORELIMBS** | Humerus | 12 | 31 |
|  | Radius | 9 |  |
|  | Ulna | 7 |  |
|  | Scapula | 3 |  |
| **PROXIMAL HINDLIMB** | Femur | 7 | 31 |
|  | Tibia | 15 |  |
|  | Patella | 3 |  |
|  | Pelvis | 6 |  |
