# Supplementary material for: Cattle on the rocks: Understanding cattle mobility, diet, and seasonality in the Iberian Peninsula. The Middle Neolithic site of Cova de les Pixarelles (Tavertet, Osona)
Source: PLoS One. 2025 Jan 27;20(1):e0317723. doi: 10.1371/journal.pone.0317723 (PMC11772053; doi:10.1371/journal.pone.0317723)
Supplement: S2 Table — The table displays all the samples with collagen analysed. Samples with C:N ratios over 3.6 were discarded and were not incorporated in the baseline. (DOCX) [file pone.0317723.s005.docx]

**Cattle on the rocks: Understanding cattle mobility, diet, and seasonality in the Iberian Peninsula. The Middle Neolithic site of Cova de les Pixarelles (Tavertet, Osona)**

**Roger Alcàntara Fors^1,2*^, Richard Madgwick^1*^, Laura C. Viñas-Caron ^2,3^, Alexandra J. Nederbragt^4^, Maria Saña Seguí^1*^**

**Supporting information**

**S2 Table. Carbon and Nitrogen data prepared for this paper from the cattle recovered in the sites of Cova de les Pixarelles, Reina Amàlia and la Draga. The table displays all the samples with collagen analysed. Samples with C:N ratios over 3.6 were discarded and were not incorporated in the baseline.**

| **Site** | **Element** | **ID** | **δ13C**  **(‰ VPDB)** | **δ15N**  **(‰ AIR)** | **%C** | **%N** | **C:N** | **Preparation** | **Laboratory** |
| --- | --- | --- | --- | --- | --- | --- | --- | --- | --- |
| C. de les Pixarelles | Mandible | PIX1 | -19.9 | 4.7 | 27.6 | 9.9 | 3.26 | R. Alcàntara | Stable isotope laboratory, CU |
| C. de les Pixarelles | Mandible | PIX2 | -20.1 | 4.1 | 39.1 | 14 | 3.27 | R. Alcàntara | Stable isotope laboratory, CU |
| C. de les Pixarelles | M2 root | PIX3 | -20.3 | 4.4 | 43.9 | 15.4 | 3.33 | R. Alcàntara | Stable isotope laboratory, CU |
| C. de les Pixarelles | Mandible | PIX4 | -20.6 | 3.9 | 33.1 | 11.7 | 3.24 | R. Alcàntara | Stable isotope laboratory, CU |
| C. de les Pixarelles | Mandible | PIX5 | -19.4 | 5.1 | 36.9 | 13.1 | 3.27 | R. Alcàntara | Stable isotope laboratory, CU |
| C. de les Pixarelles | Radius | BL-PX1 | -20.73 | 4.08 | 39.97 | 13.62 | 3.4 | L. C. Viñas | ICTA - UAB |
| C. de les Pixarelles | Radius | BL-PX2 | -21.39 | 4.00 | 24.43 | 7.42 | 3.8 | L. C. Viñas | ICTA - UAB |
| C. de les Pixarelles | Metatarsal | BL-PX3 | -20.69 | 5.53 | 14.94 | 5.10 | 3.4 | L. C. Viñas | ICTA - UAB |
| C. de les Pixarelles | Tibia | BL-PX4 | -22.33 | 3.81 | 28.29 | 8.01 | 4.1 | L. C. Viñas | ICTA - UAB |
| C. de les Pixarelles | Metacarpal | BL-PX5 | -21.20 | 5.13 | 35.07 | 12.02 | 3.4 | L. C. Viñas | ICTA - UAB |
| C. de les Pixarelles | Radius | BL-PX6 | -20.39 | 3.90 | 41.05 | 14.76 | 3.2 | L. C. Viñas | ICTA - UAB |
| C. de les Pixarelles | Humerus | BL-PX7 | -21.99 | 3.81 | 34.30 | 10.59 | 3.8 | L. C. Viñas | ICTA - UAB |
| C. de les Pixarelles | Metatarsal | BL-PX9 | -21.41 | 4.38 | 32.43 | 10.60 | 3.6 | L. C. Viñas | ICTA - UAB |
| C. de les Pixarelles | Metacarpal | BL-PX12 | -22.23 | 3.63 | 33.95 | 10.03 | 3.9 | L. C. Viñas | ICTA - UAB |
| C. de les Pixarelles | Metatarsal | BL-PX13 | -20.66 | 3.74 | 37.30 | 13.09 | 3.3 | L. C. Viñas | ICTA - UAB |
| C. de les Pixarelles | Tibia | BL-PX14 | -20.36 | 3.47 | 34.05 | 12.50 | 3.2 | L. C. Viñas | ICTA - UAB |
| C. de les Pixarelles | Metatarsal | BL-PX15 | -20.83 | 4.01 | 35.94 | 12.36 | 3.4 | L. C. Viñas | ICTA - UAB |
| C. de les Pixarelles | Metacarpal | BL-PX16 | -21.11 | 3.72 | 31.14 | 10.69 | 3.4 | L. C. Viñas | ICTA - UAB |
| C. de les Pixarelles | Femur | BL-PX17 | -21.90 | 4.82 | 33.07 | 11.52 | 3.3 | L. C. Viñas | ICTA - UAB |
| C. de les Pixarelles | Tibia | BL-PX19 | -20.56 | 4.13 | 33.91 | 11.75 | 3.4 | L. C. Viñas | ICTA - UAB |
| C. de les Pixarelles | Radius | BL-PX23 | -24.05 | 4.39 | 31.88 | 6.24 | 6 | L. C. Viñas | ICTA - UAB |
| Reina Amàlia | Humerus | BL-RA1 | -21.21 | 6.56 | 32.37 | 11.77 | 3.2 | L. C. Viñas | ICTA - UAB |
| Reina Amàlia | Humerus | BL-RA2 | -20.08 | 5.15 | 24.37 | 8.83 | 3.2 | L. C. Viñas | ICTA - UAB |
| Reina Amàlia | Metacarpal | BL-RA4 | -20.48 | 6.36 | 18.09 | 6.22 | 3.4 | L. C. Viñas | ICTA - UAB |
| Reina Amàlia | Humerus | BL-RA5 | -21.25 | 5.65 | 23.34 | 6.85 | 4 | L. C. Viñas | ICTA - UAB |
| Reina Amàlia | Metacarpal | BL-RA6 | -19.89 | 5.21 | 40.21 | 13.93 | 3.4 | L. C. Viñas | ICTA - UAB |
| La Draga | Metacarpal | BL-DR1G | -18.60 | 2.81 | 37.28 | 13.51 | 3.2 | L. C. Viñas | ICTA - UAB |
| La Draga | Metacarpal | BL-DR2 | -20.24 | 5.17 | 37.87 | 13.42 | 3.3 | L. C. Viñas | ICTA - UAB |
| La Draga | Metatarsal | BL-DR4G | -20.65 | 4.23 | 37.78 | 13.87 | 3.2 | L. C. Viñas | ICTA - UAB |
| La Draga | Metacarpal | BL-DR5G | -20.52 | 5.53 | 32.23 | 11.29 | 3.3 | L. C. Viñas | ICTA - UAB |
| La Draga | Metatarsal | BL-DR7G | -19.82 | 6.15 | 35.72 | 12.94 | 3.2 | L. C. Viñas | ICTA - UAB |
| La Draga | Metatarsal | BL-DR8G | -19.22 | 6.15 | 37.06 | 13.39 | 3.2 | L. C. Viñas | ICTA - UAB |
| La Draga | Metatarsal | BL-DR9 | -20.96 | 5.40 | 36.53 | 12.57 | 3.4 | L. C. Viñas | ICTA - UAB |
| La Draga | Metacarpal | BL-DR11G | -20.46 | 5.08 | 40.83 | 14.68 | 3.2 | L. C. Viñas | ICTA - UAB |
| La Draga | Metacarpal | BL-DR12G | -21.92 | 4.47 | 35.44 | 12.44 | 3.3 | L. C. Viñas | ICTA - UAB |
| La Draga | Metatarsal | BL-DR13 | -18.00 | 5.44 | 39.16 | 14.33 | 3.2 | L. C. Viñas | ICTA - UAB |
| La Draga | Metacarpal | BL-DR14G | -20.56 | 5.30 | 37.73 | 13.64 | 3.2 | L. C. Viñas | ICTA - UAB |
| La Draga | Metatarsal | BL-DR17G | -20.47 | 5.58 | 38.49 | 13.40 | 3.3 | L. C. Viñas | ICTA - UAB |
| La Draga | Metatarsal | BL-DR18 | -20.53 | 4.98 | 38.47 | 14.00 | 3.2 | L. C. Viñas | ICTA - UAB |
| La Draga | Metatarsal | BL-DR20 | -17.52 | 5.31 | 36.15 | 13.39 | 3.1 | L. C. Viñas | ICTA - UAB |
| La Draga | Metacarpal | BL-DR23G | -19.73 | 5.33 | 37.36 | 13.18 | 3.3 | L. C. Viñas | ICTA - UAB |
| La Draga | Metatarsal | BL-DR24 | -21.30 | 4.89 | 36.90 | 12.78 | 3.4 | L. C. Viñas | ICTA - UAB |
| La Draga | Metacarpal | BL-DR25G | -21.27 | 4.76 | 34.47 | 12.13 | 3.3 | L. C. Viñas | ICTA - UAB |
