# Supplementary material for: Cattle on the rocks: Understanding cattle mobility, diet, and seasonality in the Iberian Peninsula. The Middle Neolithic site of Cova de les Pixarelles (Tavertet, Osona)
Source: PLoS One. 2025 Jan 27;20(1):e0317723. doi: 10.1371/journal.pone.0317723 (PMC11772053; doi:10.1371/journal.pone.0317723)
Supplement: S3 Table — Distance of the samples from the Enamel-Root Junction (ERJ dist.) is expressed in millimetres. δ13C and δ18O values are expressed in ‰ and corrected using the Vienna Peedee Belemnite (VPDB) standard. (DOCX) [file pone.0317723.s006.docx]

**Cattle on the rocks: Understanding cattle mobility, diet, and seasonality in the Iberian Peninsula. The Middle Neolithic site of Cova de les Pixarelles (Tavertet, Osona)**

**Roger Alcàntara Fors^1,2*^, Richard Madgwick^1*^, Laura C. Viñas-Caron ^2,3^, Alexandra J. Nederbragt^4^, Maria Saña Seguí^1*^**

**Supporting information**

**S3 Table. Cattle teeth enamel sequential Carbon and Oxygen results from Cova de les Pixarelles. Distance of the samples from the Enamel-Root Junction (ERJ dist.) is expressed in millimetres. δ13C and δ18O values are expressed in ‰ and corrected using the Vienna Peedee Belemnite (VPDB) standard.**

| **M2** | **PIX 1** | **Sample** | **ERJ dist.** | **δ13C** | **δ18O** | **PIX 2** | **Sample** | **ERJ dist.** | **δ13C** | **δ18O** | **PIX 3** | **Sample** | **ERJ dist.** | **δ13C** | **δ18O** | **PIX 4** | **Sample** | **ERJ dist.** | **δ13C** | **δ18O** | **PIX 5** | **Sample** | **ERJ dist.** | **δ13C** | **δ18O** |
| --- | --- | --- | --- | --- | --- | --- | --- | --- | --- | --- | --- | --- | --- | --- | --- | --- | --- | --- | --- | --- | --- | --- | --- | --- | --- |
|  |  | PX1 M2.1 | 34.7 | -10.8 | -6.1 |  | PX2 M2.1 | 18.7 | -10.7 | -5.7 |  | PX3 M2.1 | 31.8 | -12.3 | -4.9 |  | PX4 M2.1 | 25.2 | -11.4 | -5.9 |  | PX5 M2.1 | 14.6 | -9.9 | -4.0 |
|  |  | PX1 M2.2 | 30.4 | -11.1 | -5.9 |  | PX2 M2.2 | 17.2 | -10.6 | -5.7 |  | PX3 M2.2 | 30.1 | -12.3 | -4.7 |  | PX4 M2.2 | 22.8 | -11.5 | -6.3 |  | PX5 M2.2 | 13.1 | -10.1 | -4.8 |
|  |  | PX1 M2.3 | 28.2 | -10.8 | -6.1 |  | PX2 M2.3 | 15.8 | -10.7 | -5.9 |  | PX3 M2.3 | 28.1 | -11.8 | -5.1 |  | PX4 M2.3 | 20.9 | -11.6 | -5.6 |  | PX5 M2.3 | 10.8 | -10.5 | -5.1 |
|  |  | PX1 M2.4 | 26.0 | -11.2 | -5.8 |  | PX2 M2.4 | 14.5 | -10.6 | -5.7 |  | PX3 M2.4 | 26.4 | -11.8 | -5.6 |  | PX4 M2.4 | 18.3 | -11.4 | -6.0 |  | PX5 M2.4 | 9.0 | -10.9 | -5.8 |
|  |  | PX1 M2.5 | 23.7 | -10.7 | -5.9 |  | PX2 M2.5 | 13.2 | -10.5 | -5.9 |  | PX3 M2.5 | 24.7 | -11.9 | -5.7 |  | PX4 M2.5 | 16.9 | -11.6 | -5.7 |  | PX5 M2.5 | 6.7 | -11.3 | -5.9 |
|  |  | PX1 M2.6 | 21.9 | -10.9 | -5.6 |  | PX2 M2.6 | 12.0 | -10.8 | -5.9 |  | PX3 M2.6 | 22.9 | -11.7 | -5.9 |  | PX4 M2.6 | 14.5 | -11.4 | -6.1 |  | PX5 M2.6 | 4.6 | -10.2 | -5.7 |
|  |  | PX1 M2.7 | 19.2 | -11.0 | -5.5 |  | PX2 M2.7 | 10.4 | -10.7 | -5.6 |  | PX3 M2.7 | 21.2 | -11.6 | -5.9 |  | PX4 M2.7 | 12.6 | -11.6 | -5.4 |  | PX5 M2.7 | 2.1 | -11.4 | -5.4 |
|  |  | PX1 M2.8 | 16.9 | -11.0 | -5.8 |  | PX2 M2.8 | 8.9 | -11.0 | -6.4 |  | PX3 M2.8 | 19.6 | -11.4 | -6.3 |  | PX4 M2.8 | 10.0 | -11.6 | -6.1 |  | PX5 M2.8 | 0.3 | -11.4 | -5.1 |
|  |  | PX1 M2.9 | 14.5 | -10.6 | -6.1 |  | PX2 M2.9 | 7.0 | -10.9 | -5.7 |  | PX3 M2.9 | 17.8 | -11.4 | -6.4 |  | PX4 M2.9 | 8.5 | -11.4 | -5.2 |  |  |  |  |  |
|  |  | PX1 M2.10 | 12.7 | -10.8 | -6.0 |  | PX2 M2.10 | 5.4 | -11.1 | -5.5 |  | PX3 M2.10 | 16.1 | -11.2 | -7.1 |  | PX4 M2.10 | 5.8 | -11.2 | -6.1 |  |  |  |  |  |
|  |  | PX1 M2.11 | 10.2 | -10.5 | -5.8 |  | PX2 M2.11 | 4.0 | -10.9 | -5.3 |  | PX3 M2.11 | 14.6 | -11.3 | -7.1 |  | PX4 M2.11 | 3.8 | -11.4 | -5.7 |  |  |  |  |  |
|  |  | PX1 M2.12 | 8.3 | -10.8 | -6.2 |  | PX2 M2.12 | 2.5 | -11.0 | -5.3 |  | PX3 M2.12 | 12.7 | -11.1 | -7.4 |  | PX4 M2.12 | 1.3 | -11.0 | -6.0 |  |  |  |  |  |
|  |  | PX1 M2.13 | 5.9 | -10.5 | -6.4 |  | PX2 M2.13 | 1.0 | -10.9 | -5.2 |  | PX3 M2.13 | 11.3 | -10.9 | -7.2 |  | PX4 M2.13 | 0.1 | -11.0 | -5.9 |  |  |  |  |  |
|  |  | PX1 M2.14 | 4.0 | -10.7 | -6.4 |  |  |  |  |  |  | PX3 M2.14 | 9.5 | -11.2 | -7.4 |  |  |  |  |  |  |  |  |  |  |
|  |  | PX1 M2.15 | 1.2 | -9.9 | -5.7 |  |  |  |  |  |  | PX3 M2.15 | 7.9 | -10.7 | -7.3 |  |  |  |  |  |  |  |  |  |  |
|  |  |  |  |  |  |  |  |  |  |  |  | PX3 M2.16 | 6.3 | -11.1 | -7.6 |  |  |  |  |  |  |  |  |  |  |
|  |  |  |  |  |  |  |  |  |  |  |  | PX3 M2.17 | 4.6 | -11.0 | -7.0 |  |  |  |  |  |  |  |  |  |  |
|  |  |  |  |  |  |  |  |  |  |  |  | PX3 M2.18 | 3.0 | -10.9 | -6.9 |  |  |  |  |  |  |  |  |  |  |
|  |  |  |  |  |  |  |  |  |  |  |  | PX3 M2.19 | 1.1 | -10.9 | -6.7 |  |  |  |  |  |  |  |  |  |  |
| **M3** |  | **Sample** | **ERJ dist.** | **δ13C** | **δ18O** |  | **Sample** | **ERJ dist.** | **δ13C** | **δ18O** |  | **Sample** | **ERJ dist.** | **δ13C** | **δ18O** |  | **Sample** | **ERJ dist.** | **δ^13^C** | **δ^18^O** |  | **Sample** | **ERJ dist.** | **δ13C** | **δ18O** |
|  |  | PX1 M3.1 | 36.2 | -11.1 | -7.1 |  | PX2 M3.1 | 27.9 | -10.9 | -5.6 |  | PX3 M3.1 | 32.9 | -10.6 | -6.1 |  | PX4 M3.1 | 35.1 | -11.3 | -6.2 |  | PX5 M3.1 | 28.4 | -10.8 | -4.6 |
|  |  | PX1 M3.2 | 34.6 | -11.0 | -5.8 |  | PX2 M3.2 | 26.1 | -10.7 | -5.4 |  | PX3 M3.2 | 31.4 | -10.8 | -5.7 |  | PX4 M3.2 | 33.5 | -11.3 | -5.8 |  | PX5 M3.2 | 25.8 | -10.7 | -5.0 |
|  |  | PX1 M3.3 | 32.2 | -10.7 | -6.1 |  | PX2 M3.3 | 24.4 | -10.7 | -6.1 |  | PX3 M3.3 | 29.0 | -10.4 | -5.5 |  | PX4 M3.3 | 31.3 | -11.2 | -5.8 |  | PX5 M3.3 | 23.9 | -10.3 | -4.9 |
|  |  | PX1 M3.4 | 30.4 | -11.2 | -5.6 |  | PX2 M3.4 | 23.0 | -10.7 | -5.3 |  | PX3 M3.4 | 27.2 | -11.1 | -5.6 |  | PX4 M3.4 | 29.3 | -11.2 | -5.7 |  | PX5 M3.4 | 22.0 | -10.4 | -4.5 |
|  |  | PX1 M3.5 | 27.9 | -11.0 | -5.9 |  | PX2 M3.5 | 21.4 | -10.4 | -6.2 |  | PX3 M3.5 | 24.9 | -10.8 | -5.6 |  | PX4 M3.5 | 26.8 | -11.2 | -5.6 |  | PX5 M3.5 | 20.0 | -10.2 | -4.9 |
|  |  | PX1 M3.6 | 26.2 | -10.9 | -5.5 |  | PX2 M3.6 | 19.8 | -10.5 | -5.5 |  | PX3 M3.6 | 23.3 | -10.9 | -5.7 |  | PX4 M3.6 | 24.7 | -11.1 | -5.6 |  | PX5 M3.6 | 18.2 | -10.2 | -4.8 |
|  |  | PX1 M3.7 | 23.6 | -11.0 | -6.6 |  | PX2 M3.7 | 18.6 | -10.2 | -6.2 |  | PX3 M3.7 | 21.3 | -10.9 | -6.0 |  | PX4 M3.7 | 22.2 | -11.2 | -5.3 |  | PX5 M3.7 | 16.3 | -9.9 | -4.4 |
|  |  | PX1 M3.8 | 22.1 | -10.9 | -6.1 |  | PX2 M3.8 | 17.1 | -10.4 | -5.8 |  | PX3 M3.8 | 19.3 | -10.9 | -6.1 |  | PX4 M3.8 | 19.9 | -11.2 | -5.0 |  | PX5 M3.8 | 14.3 | -9.9 | -4.5 |
|  |  | PX1 M3.9 | 19.7 | -10.5 | -6.2 |  | PX2 M3.9 | 15.4 | -10.5 | -6.3 |  | PX3 M3.9 | 17.4 | -10.6 | -6.4 |  | PX4 M3.9 | 17.8 | -11.0 | -4.8 |  | PX5 M3.9 | 12.1 | -10.2 | -4.5 |
|  |  | PX1 M3.10 | 17.6 | -10.6 | -6.2 |  | PX2 M3.10 | 13.9 | -10.6 | -6.4 |  | PX3 M3.10 | 15.3 | -10.6 | -6.6 |  | PX4 M3.10 | 15.8 | -11.1 | -5.1 |  | PX5 M3.10 | 10.3 | -10.5 | -4.6 |
|  |  | PX1 M3.11 | 15.1 | -10.4 | -6.7 |  | PX2 M3.11 | 12.4 | -10.5 | -6.9 |  | PX3 M3.11 | 13.2 | -10.4 | -6.9 |  | PX4 M3.11 | 13.8 | -11.1 | -4.7 |  | PX5 M3.11 | 8.4 | -10.6 | -5.0 |
|  |  | PX1 M3.12 | 13.2 | -10.7 | -6.8 |  | PX2 M3.12 | 11.1 | -10.6 | -6.5 |  | PX3 M3.12 | 11.4 | -10.5 | -6.6 |  | PX4 M3.12 | 12.0 | -11.1 | -5.4 |  | PX5 M3.12 | 6.5 | -11.3 | -5.1 |
|  |  | PX1 M3.13 | 10.7 | -10.4 | -7.2 |  | PX2 M3.13 | 9.7 | -10.6 | -6.6 |  | PX3 M3.13 | 9.4 | -10.5 | -7.1 |  | PX4 M3.13 | 9.4 | -10.7 | -5.6 |  | PX5 M3.13 | 4.6 | -11.7 | -5.6 |
|  |  | PX1 M3.14 | 8.8 | -10.6 | -7.2 |  | PX2 M3.14 | 8.1 | -10.8 | -6.5 |  | PX3 M3.14 | 7.2 | -10.6 | -7.1 |  | PX4 M3.14 | 7.7 | -10.9 | -5.9 |  | PX5 M3.14 | 2.4 | -11.2 | -4.7 |
|  |  | PX1 M3.15 | 6.5 | -10.0 | -7.1 |  | PX2 M3.15 | 6.5 | -10.3 | -6.2 |  | PX3 M3.15 | 5.4 | -10.0 | -7.3 |  | PX4 M3.15 | 5.4 | -10.9 | -5.5 |  | PX5 M3.15 | 0.6 | -11.3 | -4.7 |
|  |  | PX1 M3.16 | 4.5 | -10.4 | -7.1 |  | PX2 M3.16 | 5.1 | -10.8 | -5.9 |  | PX3 M3.16 | 3.8 | -10.6 | -7.9 |  | PX4 M3.16 | 3.7 | -9.7 | -5.0 |  |  |  |  |  |
|  |  | PX1 M3.17 | 1.9 | -10.0 | -6.7 |  | PX2 M3.17 | 3.7 | -10.7 | -5.2 |  | PX3 M3.17 | 1.8 | -10.1 | -7.1 |  | PX4 M3.17 | 1.4 | -10.7 | -5.8 |  |  |  |  |  |
|  |  | PX1 M3.18 | 0.1 | -10.5 | -6.3 |  | PX2 M3.18 | 2.4 | -10.9 | -4.9 |  |  |  |  |  |  |  |  |  |  |  |  |  |  |  |
|  |  |  |  |  |  |  | PX2 M3.19 | 1.0 | -10.6 | -4.7 |  |  |  |  |  |  |  |  |  |  |  |  |  |  |  |
